# Chemical and Pharmacological Potential of *Coccoloba cowellii*, an Endemic Endangered Plant from Cuba

**DOI:** 10.3390/molecules26040935

**Published:** 2021-02-10

**Authors:** Daniel Méndez, Julio C. Escalona-Arranz, Kenn Foubert, An Matheeussen, Anastasia Van der Auwera, Stefano Piazza, Ann Cuypers, Paul Cos, Luc Pieters

**Affiliations:** 1 Chemistry Department, Faculty of Applied Sciences, University of Camagüey, Carretera de Circunvalación Km 5 ½, Camagüey 74650, Cuba; daniel.mendez@reduc.edu.cu; 2 Pharmacy Department, Faculty of Natural and Exact Sciences, Oriente University, Avenida Patricio Lumumba s/n, Santiago de Cuba 90500, Cuba; jcea@uo.edu.cu; 3 Natural Products & Food Research and Analysis (NatuRA), Department of Pharmaceutical Sciences, University of Antwerp, Universiteitsplein 1, BE-2610 Antwerp, Belgium; kenn.foubert@uantwerpen.be (K.F.); anastasia.vanderauwera@uantwerpen.be (A.V.d.A.); 4 Laboratory of Microbiology, Parasitology and Hygiene (LMPH), Faculty of Pharmaceutical, Biomedical and Veterinary Sciences, University of Antwerp, Universiteitsplein 1, BE-2610 Antwerp, Belgium; an.matheeussen@uantwerpen.be; 5 Laboratory of Pharmacognosy, Department of Pharmacological and Biomolecular Sciences, University of Milan/UNIMI, IT-20133 Milan, Italy; stefano.piazza@unimi.it; 6 Centre for Environmental Sciences, Campus Diepenbeek, Hasselt University, Agoralaan Building D, BE-3590 Diepenbeek, Belgium; ann.cuypers@uhasselt.be

**Keywords:** *Coccoloba cowellii*, endemic plant, UHPLC-ESI-QTOF-MS, flavonoids, antifungal, antibacterial, COX-1/2 inhibition

## Abstract

*Coccoloba cowellii* Britton (Polygonaceae) is an endemic and critically endangered plant that only grows in Camagüey, a province of Cuba. In this study, a total of 13 compounds were identified in a methanolic leaf extract, employing a dereplication of the UHPLC-HRMS data by means of feature-based molecular networking (FBMN) analysis in the Global Natural Products Social Molecular Network (GNPS), together with the interpretation of the MS/MS data and comparison with the literature. The major constituents were glucuronides and glycosides of myricetin and quercetin, as well as epichatechin-3-*O*-gallate, catechin, epicatechin and gallic acid, all of them being reported for the first time in *C. cowellii* leaves. The leaf extract was also tested against various microorganisms, and it showed a strong antifungal effect against *Candida albicans* ATCC B59630 (azole-resistant) (IC_50_ 2.1 µg/mL) and *Cryptococcus neoformans* ATCC B66663 (IC_50_ 4.1 µg/mL) with no cytotoxicity (CC_50_ > 64.0 µg/mL) on MRC-5 SV2 cells, determined by the resazurin assay. Additionally, the extract strongly inhibited COX-1 and COX-2 enzyme activity using a cell-free experiment in a dose-dependent manner, being significantly more active on COX-1 (IC_50_ 4.9 µg/mL) than on COX-2 (IC_50_ 10.4 µg/mL). The constituents identified as well as the pharmacological activities measured highlight the potential of *C. cowellii* leaves, increasing the interest in the implementation of conservation strategies for this species.

## 1. Introduction

All over the world, plant biodiversity is at risk, and every year, the number of threatened species increases dramatically [1]. Endemic plant species are usually more vulnerable to anthropogenic threats and natural changes and, therefore, hold a higher extinction risk when no management actions are designed. The exploration in such species of their phytochemical and/or pharmacological profiles might provide leads toward the discovery of new compounds and/or biological activities. In consequence, conferring an economic value to these species, not only because of the presence of valuable pharmacologically active compounds, but also others with industrial potential such as biofuel production and bioremediation, could increase the interest in such species and encourage the implementation of conservation strategies [2].

Cuba is recognized as the island with the highest degree of endemism in the West-Indies, including more than half of its plant species [3]. Since plants in Cuba are frequently subjected to harsh environmental conditions (e.g., high temperature, drought, high levels of sunlight, salinity, nutrient-poor soil conditions), combined with the fact that the Cuban flora is pharmacologically and chemically under-investigated [4], the development of conservation strategies to preserve the plant species of the island is necessary.

The genus *Coccoloba* comprises approximately 120–150 species of flowering plants from the subfamily Erigonoideae of the Polygonaceae family, order Caryophyllales. It is native to the tropical and subtropical regions of America, i.e., South America, the Caribbean and Central America, with two species that extend to Florida [5]. A small number of species of *Coccoloba* are used in traditional medicine in tropical and subtropical regions of the Americas related to the treatment of several ailments, as an astringent, analgesic and anesthetic, for the treatment of fever and diarrhea, menstrual disturbance, uterine hemorrhages, hemorrhoids and gonorrhea [6,7,8]. The phytochemistry of the genus has not been widely explored, and most of the studies are centered on the more common species *C. uvifera* (sea grape, native to coastal beaches throughout tropical America and the Caribbean). The characterization of few members of this genus shows a high chemical diversity of metabolite groups such as flavonoids and tannins [9,10,11,12], terpenoids and sterols [9,13,14,15], anthraquinones [9,14,16] and volatile oils [17].

Further, some biological activities have been reported for *Coccoloba* species associated with their popular uses and identified constituents. The ethyl acetate partition of the methanol extract of *C. uvifera* seeds exhibited antibacterial activity against the Gram-negative bacteria *Pseudomonas aeruginosa* and *Escherichia coli*, in addition to antifungal activity against *Fusarium oxysporum*, *Candida albicans* and *Fusarium decencellulare* [18]. The ethanol extract of *C. acrostichoides* aerial parts shows activity against *Staphylococcus aureus* and *Micrococcus luteus*. Most of the fractions, especially the *n*-hexane and ethyl acetate fractions, also had an antifungal activity [13]. The ethanolic bark extract of *C. dugandiana* exhibited an inhibitory effect on the growth of *Cryptococcus neoformans* [11], while the crude leaf extract of *C. parimensis* revealed an antiplasmodial activity through a DNA-based microfluorimetric method [19].

In Cuba, the presence of 34 species of *Coccoloba* has been reported [20]. From them, 25 are recognized as endemic. Nevertheless, only information about the ethnopharmacological use of *C. uvifera* was found in the literature, related to the treatment of sores and grains, hoarseness, asthma, dysentery and body itching and its use as an anti-hemorrhagic [21]. One of the almost unknown endemic species of this genus that grows in Cuba is *Coccoloba cowellii* Britton, which classifies as critically endangered (CR) according to the International Union for Conservation of Nature (IUCN) [22]. Only preliminary information is available about its phytochemical composition and its antioxidant activity [23]. This gap in the knowledge of this species, which risks disappearing without having been explored in more detail, has initiated this research. For this purpose, ultrahigh-performance liquid chromatography-high-resolution mass spectrometry (UHPLC-HRMS) was selected as an analytical technique suitable for studying the non-volatile phytochemical composition of *C. cowellii* leaves, collecting as little plant material as possible. Microanalytical pharmacological tests were also considered with this conservation purpose.

## 2. Results

### 2.1. UHPLC-HRMS Analysis

**Figure 1:**
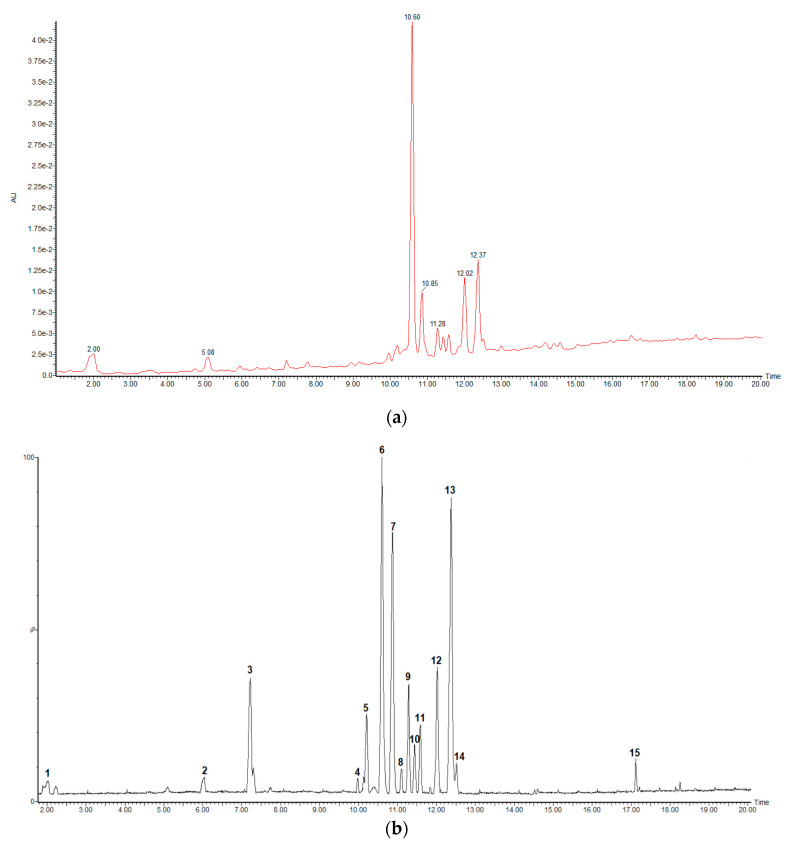
HPLC-DAD/QTOF-MS chromatograms of the 80% methanol extract of *C. cowellii* leaves: (**a**) UV detection at 280 nm and (**b**) base peak intensity (BPI) chromatogram (negative ion mode).

In the present work, a qualitative analysis of the chemical composition of *C. cowellii* leaves was carried out using UHPLC-UV-QTOF-ESI-MS in negative ionization mode. Figure 1 shows the base peak intensity (BPI, peaks 1 to 15 corresponding to Table 1) chromatogram at 280 nm (a) and in MS negative ionization mode (b) of *C. cowellii* leaf extract. From the peak intensity of the UV chromatogram (Figure 1a), it is inferred that compound 6 (Rt = 10.60 min) appears as the main compound. Peaks 7, 12 and 13 (Rt = 10.85, 12.02 and 12.37) also reach high concentration ratios regarding the rest of the compounds.

A dereplication strategy was used to analyze the raw data obtained. Dereplication provides fast identification of known metabolites in complex biological mixtures using small quantities of material, speeding up the discovery of novel natural products [24]. Feature-based molecular networking (FBMN), available on the Global Natural Products Social Molecular Networking (GNPS) web platform at https://gnps.ucsd.edu (accessed on 16 January 2021), is ideally suited for advanced molecular networking analysis, enabling the characterization of isomers, relative quantification and the integration of ion mobility data. That is why FBMN is considered the recommended way to analyze single LC-MS^2^ metabolomics data [25]. With this purpose, the spectra in the network were searched and matched with GNPS spectral libraries, rendering 12 library hits (Table S1, Supplementary Materials). The matched compounds were mainly glycosides and glucuronides of the aglycones quercetin and myricetin, proanthocyanidins and one methoxylated flavonoid.

Later on, all the major peaks detected were tentatively characterized by means of MS data, together with the interpretation of the observed MS/MS spectra in comparison with those found in the literature and the information derived from the FBMN analysis previously conducted. The formerly identified phytochemicals from the same botanical family or species were also utilized in the identification when applicable. This analysis allowed the identification of 13 phytochemical compounds from a total of 15 peaks. Four compounds were confirmed using authentic standards, while the others were tentatively characterized. All of them were reported for the first time in *C. cowellii* leaves (Table 1).

The fragment nomenclature employed for flavonoid glycosides was applied according to Vukics and Guttman [26]. The MS spectra are shown in the Supplementary Materials (Figure S2, a–o).

Peak 1 was identified and confirmed using the chemically pure standard gallic acid, with an MW of 170, and an ion at *m*/*z* 169 [M − H]^−^, yielding a fragment at *m*/*z* 125 due to the loss of CO_2_ (44 Da). Peaks 2 and 3 both presented an [M − H]^−^ ion at *m*/*z* 289. Comparing the results with the standards used, peak 2 was identified as catechin and peak 3 was identified as epicatechin.

Peaks 4, 6 and 9 all presented fragments at *m*/*z* 317 in the MS/MS spectra, suggesting the presence of the aglycone myricetin, coincident with the information derived from the FBMN study and the reports of its presence in other *Coccoloba* species. Peak 4 presented an [M − H]^−^ ion at *m*/*z* 479 and produced the most prominent ions at *m*/*z* 317 [Y_0_]^−^ (27% relative abundance, heterolytic loss of the sugar) and 316 [Y_0_-H]^−^ (100% relative abundance, homolytic loss of the sugar) and secondary fragmentations at *m*/*z* 287 [Y_0_-H-CO-H]^−^ and 271 [Y_0_-CO-H_2_O]^−^, typical of flavon-3-*O*-monoglycosides [27]. Such fragments implied the presence of myricetin-3-*O*-hexoside. On the other hand, the FBMN analysis shows a cosine score of 0.93, nine matched peaks and a low error, suggesting the presence of myricetin-3-*O*-galactoside. Peak 6 (most abundant compound in the sample) presented an [M − H]^−^ ion at *m*/*z* 493 and produced the most prominent ion at *m*/*z* 317 [Y_0_]^−^ and secondary fragmentations at *m*/*z* 287 [Y_0_-H-CO-H]^−^, 179 (1,2A^−^) and 151 (1,3A^−^) (typical retro Diels–Alder (RDA) fragmentation of flavon-3-ols having a dihydroxylated A-ring and trihydroxylated B-ring [28]). Such fragments implied the presence of myricetin-*O*-glucuronide. Peak 9 presented an [M − H]^−^ ion at *m*/*z* 463 and produced the most prominent ion at *m*/*z* 317 [Y_0_]^−^ (30% relative abundance, heterolytic loss of the sugar) and 316 [Y_0_-H]^−^ (100% relative abundance, homolytic loss of the sugar) and secondary fragmentations at *m*/*z* 287 [Y_0_-H-CO-H]^−^ and *m*/*z* 271 [Y_0_-CO-H_2_O]^−^, typical of flavon-3-*O*-monoglycosides [27]. Such fragments implied the presence of myricetin-3-*O*-deoxyhexoside.

Peak 5 presented an [M − H]^−^ ion at *m*/*z* 729, yielding fragments at *m*/*z* 577 [M − 152 − H]^−^ (loss of a galloyl fragment), *m*/*z* 451 due to a heterocyclic ring fission (HRF), *m*/*z* 441 (upper part of the quinone methide (QM) fission of the [M − H]^−^ ion), *m*/*z* 407 (water loss from the retro Diels–Alder (RDA) fragmentation of the *m*/*z* 577 ion) and *m*/*z* 289 and *m*/*z* 287 (upper and lower part, respectively, of the quinone methide (QM) fission of the *m*/*z* 577 ion) [29]. Such fragments implied the presence of a B-type procyanidin, monogallate. The FBMN analysis reports a peak of *m*/*z* 577, with a cosine score of 0.81, eleven matched peaks and a low error, suggesting the presence of procyanidin B1 monogallate.

Peak 7 presented an [M − H]^−^ ion at *m*/*z* 441, yielding fragments at *m*/*z* 289 [M − 152 − H]^−^ (loss of a galloyl fragment), *m*/*z* 169 (gallic acid fragment) and *m*/*z* 125 (due to the loss of CO_2_ (44 Da) from the gallic acid fragment). Such fragments implied the presence of (epi)catechin-*O*-gallate. Comparing the results with the standards, peak 7 was identified as epicatechin-3-*O*-gallate.

Peaks 10 to 14 all presented a base peak at *m*/*z* 301 in the MS/MS spectra attributed to quercetin derivates considering the information from the FBMN study, the further MS^2^ fragmentation pattern and the informs of its presence in other *Coccoloba* species. Peaks 10 and 11 presented an [M − H]^−^ ion at *m*/*z* 463 and produced the most prominent ions at *m*/*z* 301 [Y_0_]^−^ (53% and 65% relative abundance, respectively, heterolytic loss of the sugar) and 300 [Y_0_-H]^−^ (100% relative abundance, homolytic loss of the sugar) and a secondary fragment at *m*/*z* 271 [Y_0_-H-CO-H]^−^. Such fragments implied the presence of quercetin-*O*-hexoside, but due the similarity of the spectra, it was impossible to differentiate between possible isomers; therefore, they were assigned as quercetin-*O*-hexoside 1 and 2, respectively. The FBMN analysis, with a cosine score of 0.80 and seven matched peaks, suggests the quercetin-3-*O*-galactoside presence as one of the isomers. Peak 12 presented an [M − H]^−^ ion at *m*/*z* 477 with the most prominent ion at *m*/*z* 301 [Y_0_]^−^ and a secondary fragment at *m*/*z* 271 [Y_0_-H-CO-H]^−^. Such fragments implied the presence of quercetin-*O*-glucuronide. Peaks 13 (second most abundant in the extract) and 14 presented an [M − H]^−^ ion at *m*/*z* 433 and produced the most prominent ions at *m*/*z* 301 [Y_0_]^−^ (93% and 36% relative abundance, respectively, homolytic loss of the sugar) and 300 [Y_0_-H]^−^ (100% relative abundance, homolytic loss of the sugar) and secondary fragments at *m*/*z* 271 [Y_0_-H-CO-H]^−^ and *m*/*z* 255 [Y_0_-CO-H_2_O]^−^. Such fragments implied the presence of quercetin-*O*-pentoside, but due the similarity of the spectra, it was impossible to differentiate between possible isomers; therefore, they were assigned as quercetin-*O*-pentoside 1 and 2, respectively. The FBMN analysis, with a cosine score of 0.72, six matched peaks and a low error, suggests the possibility that at least one of the isomers is quercetin-3-*O*-arabinoside.

Flavonoids are one of the most important classes of plant secondary metabolites owing to their various biological activities. Quercetin glycosides are commonly found in the family Polygonaceae. For the genus *Coccoloba*, four flavonoid glycosides were isolated from the leaf extract of *C. uvifera* [12], i.e., myricetin-3-*O*-rhamnoside, which was also previously isolated from the leaf extracts of *C. peltate* and *C. dugandiana* [9,10], myricetin-3-*O*-glucoside, quercetin-3-*O*-rhamnoside and quercetin-3-*O*-arabinoside. Epigallocatechin gallate was isolated from *C. dungandiana* [11] and gallic acid was detected in *C. dungandiana*, *C. peltata* and *C. uvifera* [9,11,18]. These coincidences in the phytochemical composition of *C. cowelli* with its congeners, in addition to fitting into the chemotaxonomic pattern, enhance its potential as a producer of biomolecules with potential pharmacological activity.

### 2.2. Antibacterial and Cytotoxic Activity

The 80% methanolic leaf extract of *C. cowellii* was tested against a broad spectrum of microorganisms, including Gram-negative and Gram-positive bacteria, yeast, mold and protozoa. Table 2 shows the results of the in vitro antimicrobial bioassays performed. In general, the extract was not active against *Staphylococcus aureus*, *Escherichia coli* and *Aspergillus fumigatus*, at the tested concentrations (0.25 to 128 µg/mL). On the other hand, it can be noted that the extract has a strong antifungal effect against *C. albicans* and *C. neoformans* and moderate activity against parasites.

Antifungal activity has been previously reported for several *Coccoloba* species. The ethanolic bark extract of *C. dugandiana*, where (−)-epigallocatechin gallate and gallic acid were isolated through a bioassay-guided fractionation, exhibited an inhibitory effect on the growth of *C. neoformans*. Results showed that (−)-epigallocatechin gallate inhibited *C. neoformans* with IC_50_ 1.6 µg/mL and an MIC value of 12.5 µg/mL. In the same study, gallic acid was reported as inactive [11]. The antifungal activity of *C. mollis* ethanolic extracts (leaves and roots), as well as the anthraquinones isolated from the roots of this plant, was active against *Botryosphaeria rhodina*, *Botryosphaeria ribis*, *Lasiodiplodia theobromae* and *Fusarium* species (well-known fungal phytopathogens). The ethanolic extract showed promising antifungal activity, whereas the most active compound was emodin, which was able to inhibit up to 44% of the microorganism growth [16]. The ethyl acetate partition of the methanol extract *C. uvifera* seeds also exhibited antifungal activity against *Fusarium oxysporum*, *Candida albicans* and *Fusarium decencellulare* [18]. As demonstrated by these reports, the antifungal activity of *Coccoloba* spp. is mainly associated with the wide diversity of secondary metabolites, but mainly of the polyphenol type. Leaves of *C. cowellii* are rich in flavonoids and proanthocyanidins, compounds with many biological activities, including antifungal [30,31]; therefore, the antifungal activity detected in this study can be associated with the compounds identified (see Table 1).

*Coccoloba* species have been less reported with regard to their antibacterial activity, with only three hits found and tested through the old and classic disc diffusion method. The ethanol extract of *C. acrostichoides* aerial parts showed weak activity against *S. aureus* (7.17 ± 0.41 mm) and *Micrococcus luteus* (10.37 ± 0.52 mm) [13], while *C. cerifera* displayed inhibition zones of 8.33 ± 0.41 and 7.33 ± 0.52 mm for *M. luteus* and *S. aureus*, respectively [32]. Facey and collaborators in 1999 performed an antibacterial screening of plants used in Jamaican folk medicine, reporting for *C. krugiia* a weak activity against the Gram-negative bacterium *Proteus mirabilis*, with an inhibition area of 10–12 mm and moderate activity against the Gram-positive bacteria *S. aureus*, with an inhibition zone of 12–14 mm [33]. In the current research, the extract was not active against the tested strains of *E. coli* nor *S. aureus.* Due to the differences with methods and strains used in those previous reports, it is difficult to make comparisons. Even so, it is considered that the broth dilution method employed in the present study offers more reliable results than the disc diffusion assay.

The antitrypanosomal activity reported here for *C. cowellii* is rather uncommon. In a study using the stem extract of *C. pubescens* measuring the inhibitory effect on the growth of trypomastigote blood forms of *Trypanosoma brucei*, a potent activity with an IC_50_ value of 0.83 ± 83 μg/mL was observed [34]. The lack of reports on the phytochemical composition of *C. pubescens* did not allow establishing a relationship with the activity observed. In the present study, a moderate effect against *T. cruzi* and *T. brucei* is reported for the extract of *C. cowellii*. The presence of some constituents can be associated with this activity: In a recent study, gallic acid was the most active compound out of six common natural phenolic acids with an IC_50_ value of 14.2 µM. This compound caused the loss of the parasite kinetoplast and decreased the expression level of the transferrin receptor, ribonucleotide reductase and cyclin 2 genes, suggesting that gallic acid possibly exerts its effect on *T. brucei* via iron chelation leading to structural and morphological changes and arrest of the cell cycle [35]. The antiparasitic activity of catechin, epicatechin and epicatechin-3-*O*-gallate has also been reported in the literature [36,37]. All those compounds identified in the *C. cowellii* extract can contribute to the antitrypanosomal activity observed.

To evaluate the selectivity of the antimicrobial activity, the cytotoxicity on MRC-5 cells was evaluated. The total extract showed no cytotoxicity on MRC-5 cells with a CC_50_ value higher than 64 µg/mL, and therefore, according to the level of activity (IC_50_ of 1.7 ± 0.6 and 2.7 ± 2.0, respectively), it can be classified as highly selective for yeast and mold.

### 2.3. Inhibition of COX-1 and COX-2 Enzymatic Activity

Cyclo-oxygenases-1/2 catalyze the oxygenation of arachidonic acid and related polyunsaturated fatty acids to endoperoxide precursors of prostanoids. Both COX isoforms are well known to be targets for many non-steroidal anti-inflammatory compounds. COX-1 is widely distributed and constitutively expressed in most tissues where it is involved in homeostatic functions, mainly in the gastrointestinal tract. The COX-2 inducible isoform, more predominant at sites of inflammation, appears to play a key role in pathophysiologic conditions such as inflammatory disorders and has driven the therapeutic development of COX-2 inhibitors [38].

**Figure 2:**
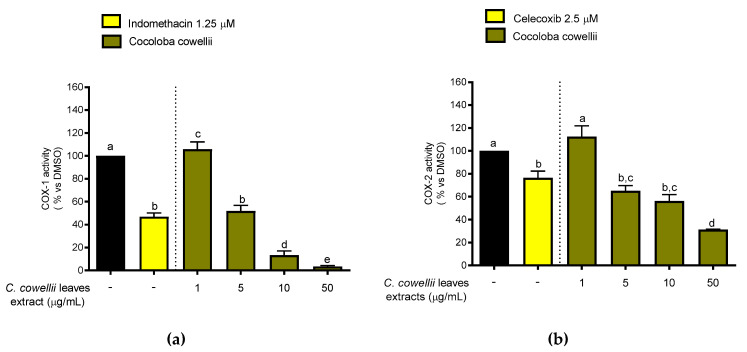
Inhibitory effect of *C. cowellii* methanolic extract on (**a**) COX-1 and (**b**) COX-2 activities. The results were analyzed using one-way ANOVA and Tukey test statistical analyses (*p*-values ≤ 0.05 were regarded as significant). Values within COX-1 and COX-2 marked with the same letter are not significantly different (at *p* ≤ 0.05).

The in vitro effect of the *C. cowellii* methanolic extract on the enzymatic activity of COX-1 and COX-2 (Figure 2) was evaluated using a cell-free experiment. This type of assay may offer prior information about the enzyme selectivity of bioactive compounds present in plant extracts. The black bars in Figure 2 represent the enzymatic activity of COXs referred to PGE_2_ production and expressed as 100%, conventionally. Lower bars from standards and the extract indicate a higher inhibition of the enzymes and therefore a better effect. The methanol extract strongly inhibited both COX-1 and COX-2 enzyme activities, in a dose-dependent manner and at non-cytotoxic concentrations, but was significantly more active on COX-1 (IC_50_ 4.9 µg/mL) than on COX-2 (IC_50_ 10.4 µg/mL). In the present study, at a concentration of 5 µg/mL, the inhibitory effects of the extract against both COX-1 and 2 are at the same level as the effect of the controls employed at their respective concentrations, indomethacin 1.25 µM and celecoxib 2.5 µM (Cayman Chemical Company, MI, USA) [39], showing the possible uses of the extract as a natural anti-inflammatory agent. Besides this good activity on both isoforms of COX enzymes, it is well known that selective inhibitors of COX-2 have created a boom in the research of new anti-inflammatory candidates in recent years. Nevertheless, it should be noticed that most of the commercial non-steroidal anti-inflammatory drugs (NSAIDs) might preferentially target COX-1 rather than COX-2, like aspirin does. Additionally, most natural products have proved to be COX-1 rather than COX-2 selective inhibitors [40].

Flavan-3-ols, widely present in wine and grapes, have shown COX-1 inhibitory effects. Catechin, epicatechin and epicatechin-3-*O*-gallate showed an IC_50_ of 1.4, 3.2 and 7.5 µM, respectively, in an assay to evaluate potential inhibition of cyclooxygenases and preneoplastic lesion formation in carcinogen-treated mouse mammary glands in organ culture [41]. These compounds can be responsible, at least in part, for the COX inhibitory effect shown by the leaves of *C. cowellii*, considering that most flavonoid derivates also show this activity.

Although COX-1 is now becoming a target to be reconsidered for cancer prevention or treatment, selective COX-1 inhibition is still a controversial issue. Inhibition of COX-1 has been strongly implicated in the gastric ulceration and bleeding induced by NSAIDs, since COX-1 is responsible for synthesis of the prostaglandins essential for normal mucosal physiology in the gut [40]. However, sometimes it is neglected that the mucosal irritation can also occur due to the acidic nature of most NSAIDs and not only by the inhibition of production of mucosal protective prostaglandins (PGEs) which leads to gastric erosion [42]. Even with the aforementioned side effects, NSAIDs represent one of the most common classes of medications used worldwide with an estimated usage of >30 million doses per day for inflammation and related disorders [43]. Further, consistent clinical studies have indicated that long-term administration of COX-2 inhibitors is associated with an enhanced risk of experiencing adverse cardiovascular events, increasing the controversial issue with regard to the selectivity of anti-inflammatory substances [44,45].

## 3. Materials and Methods

### 3.1. Chemicals and Plant Material

Solvents *n*-hexane, methanol and dimethyl sulfoxide (DMSO) were purchased from Acros Organics (Geel, Belgium) and were analytical grade, while those used for HPLC such as methanol and acetonitrile were purchased from Fisher Scientific (Leicestershire, UK). Water was dispensed by a Milli-Q system from Millipore (Bedford, MA, USA) and filtered through a 0.22 μM membrane filter before usage. Leaves of *Coccoloba cowellii* were collected near to Albaisa, in the municipality of Camagüey (Lat. 21.43615, Long. −77.83253), Cuba. The plant material was taxonomically identified by the curator of the “Julián Acuña Galé” herbarium at the University of Camagüey (HIPC, http://sweetgum.nybg.org/science/ih/herbarium-details/?irn=124935 (accessed on 16 January 2021)), where a voucher specimen was deposited (number 12057).

### 3.2. Leaf Extraction

The plant material (0.35 kg of fresh leaves), after cleaning, was dried at room temperature until constant weight and subsequently ground using a mill. The dried leaves (0.25 kg) were defatted with *n*-hexane and, later on, exhaustively stirred and macerated in 250 mL of 80% methanol/water mixture (*v*/*v*) at room temperature for five days. Every 24 h, the solvent was collected, and the material was macerated with another 250 mL. The filtrate was concentrated using a rotary evaporator under reduced pressure below 40 °C. The resulting reduced filtrate was freeze dried, yielding 25.07 g dry total extract, and stored at −20 °C until further use.

### 3.3. UHPLC-HRMS Analysis

The HPLC-DAD-QTOF analysis of the *Coccoloba cowellii* extract was carried out following the standard protocols from the Natural Products and Food Research and Analysis (NatuRA) group [46,47]. The mobile phase consisted of H_2_O + 0.1% FA (A) and ACN + 0.1% FA (B), and the gradient was set as follows (min/B%): 0.0/2.0, 1.0/2.0, 14.0/26.0, 24.0/65.0, 26.0/100.0, 29.0/100.0, 31.0/2.0, 36.0/2.0. Data were also recorded using MS^E^ in the positive and negative ionization modes (two analyses per mode), and a ramp collision energy from 10 to 30 V was applied to obtain additional structural information. Leucine-encephalin was used as the lock mass. UV detection was performed at 280 nm.

#### Data Processing

The HPLC-MS raw data were converted to abf files (Reifycs Abf Converter) and processed with MS-DIAL version 4.24 [48] for mass signal extraction between 50 and 1500 Da from 0 to 36 min. MS1 and MS2 tolerance was set at 0.01 in centroid mode. The optimized detection threshold was set to 8000 for MS1 and 5000 for MS2. The alignment results were exported using the GNPS export function of MS-DIAL.

A molecular network was created with the feature-based molecular networking (FBMN) workflow [25] on the Global Natural Products Social Molecular Networking (GNPS) web platform (https://gnps.ucsd.edu, [49] (accessed on 16 January 2021)). The precursor ion mass tolerance was set to 0.05 Da and the MS/MS fragment ion tolerance was set to 0.05 Da. The network was then created where edges were filtered to have a cosine score above 0.70 and more than 6 matched peaks (Figure S1, Supplementary Materials). Further, edges between two nodes were kept in the network if and only if each of the nodes appeared in each other’s respective top 10 most similar nodes. The spectra in the network were then searched against GNPS spectral libraries [49,50]. The library spectra were filtered in the same manner as the input data.

In the identification process, the following public databases were consulted: PubChem (https://pubchem.ncbi.nlm.nih.gov/ (accessed on 16 January 2021)), ChemSpider (https://www.chemspider.com/ (accessed on 16 January 2021)), MassBank of North America (MoNA) (http://mona.fiehnlab.ucdavis.edu/ (accessed on 16 January 2021)) and NIST Mass Spectrometry Data Center (http://chemdata.nist.gov/ (accessed on 16 January 2021)).

### 3.4. Antimicrobial Assay

The level of antimicrobial activity was arbitrarily ranked according to the following criteria: strong (IC_50_ ≤ 10 μg/mL); good (10 μg/mL< IC_50_ ≤ 20 μg/mL); moderate (20 μg/mL< IC_50_ ≤ 40 μg/mL); weak (40 μg/mL< IC_50_ ≤ 64 μg/mL); inactive (IC_50_ ≥ 64 μg/mL).

#### 3.4.1. Microorganisms and Dilutions

All microorganisms were obtained from the culture collection of the Laboratory of Microbiology, Parasitology and Hygiene (LMPH) at the University of Antwerp. *Trypanosoma cruzi* (Tulahuen CL2, β-galactosidase strain (nifurtimox-sensitive), *Trypanosoma brucei brucei* Squib 427 strain (suramin-sensitive), *Staphylococcus aureus* ATCC 6538, *Escherichia coli* ATCC8739, *Aspergillus fumigatus* ATCC B42928, *Candida albicans* ATCC B59630 (azole-resistant) and *Cryptococcus neoformans* ATCC B66663 were used for in vitro antimicrobial activity screening. The culture of the microorganisms and the sample dilutions were conducted following the protocols from the Laboratory of Microbiology, Parasitology and Hygiene (LMPH) [51,52]. The final concentration range of the samples tested was settled from 0.25 to 128 μg/mL.

#### 3.4.2. Antibacterial and Antifungal Assay

For the antibacterial and antifungal assay, the microdilution method with resazurin (redox indicator) in sterile 96-well microplates was performed according to the protocols from the Laboratory of Microbiology, Parasitology and Hygiene (LMPH) [51,52]. Doxycycline, flucytosine, econazole and miconazole were used as positive controls.

#### 3.4.3. Antitrypanosomal Assay

The *T. brucei brucei* assay was performed in 96-well microplates according to Díaz et al., 2019 [52]. The results are expressed as % reduction in parasite growth/viability compared to control wells, and the IC_50_ was calculated. Suramine was used as positive control.

For the *T. cruzi* assay, the protocol from Buckner et al., 1996 [53], was followed. The results are expressed as % reduction in parasite burdens compared to control wells, and the IC_50_ was calculated. Benznidazole was used as positive control [51].

### 3.5. Cytotoxicity Assay

MRC-5 SV2 (human fetal lung fibroblasts) cells were purchased from ATCC (American Type Culture Collection, Manassas, VA, USA). The culture conditions and the assay were conducted following the protocols from the Laboratory of Microbiology, Parasitology and Hygiene (LMPH) [51,52]. The results are expressed as % reduction in cell growth/viability compared to control wells, and the 50% cytotoxic concentration (CC_50_) was determined. Tamoxifen was used as positive control.

### 3.6. COX-1 and COX-2 Enzymatic Inhibition Assay

COX-1 and COX-2 inhibition assays were performed in a 96-well plate as previously described by Fiebich et al. [39] and Berenguer-Rivas et al., 2021 [54]. Indomethacin (1.25 µM in absolute EtOH) and celecoxib (2.5 µM in DMSO) were used as positive controls.

The concentration of PGE_2_, the main arachidonic acid metabolite in this reaction, was measured by a competitive PGE_2_ Enzyme Immunoassay (EIA) kit (Cayman Chemical Company, MI, USA). The EIA was evaluated by a microplate reader (Tecan) and the PGE_2_ concentration was determined as previously described [39,54]. Inhibition of COX refers to reduction in PGE_2_ formation in comparison to a blank run without a sample or standard.

### 3.7. Statistical Analysis

Statistical analysis was carried out in GraphPad Prism 8 (GraphPad Software, San Diego, CA, USA). All results were statistically analyzed and expressed as the means ± standard deviation (SD). The one-way analysis of variance (ANOVA) test followed by the Tukey multiple comparisons test was applied to determine the significance of differences between groups. Differences at *p* ≤ 0.05 were accepted as significant. All experiments were carried out at least twice.

## 4. Conclusions

Using UHPLC-ESI-QTOF-MS and supported by FBMN analysis, thirteen metabolites were detected from the leaves of the endemic Cuban plant *Coccoloba cowellii*. Compounds such as gallic acid, catechin, epicatechin and epicatechin-3-*O*-gallate were confirmed using standards. Quercetin and myricetin derivatives (which represent the majority in the extracts) were highlighted by the FBMN analysis. The UV spectra signed myricetin-*O*-glucuronide and quercetin-3-*O*-glucuronide as the most abundant compounds.

*C. cowellii* showed a strong antifungal activity specifically against *C. albicans* and *C. neoformans* with IC50 values below 3 µg/mL and an inhibitory effect of the enzymes COX-1 and COX-2 that, at the higher concentrations (50 µg/mL), exceeds the activity of the positive controls: indomethacin and celecoxib, respectively. These findings reveal the potential of *C. cowellii* species in the design and development of future research using this plant and, consequently, encourage the implementation of succeeding conservation strategies.

## Figures and Tables

**Table 1:** Chemical composition of the total extract from the leaves of *C. cowellii*.

Peak No.	Rt (min)	Measured Mass (*m*/*z*)	Theoretical Mass (*m*/*z*)	Accuracy (ppm)	MS/MS Ions	MF	Tentative Identification
1	2.03	169.0130	169.0137	−4.1	125.0268	C_7_H_5_O_5_	Gallic acid (std)
2	6.04	289.0728	289.0712	5.5	125.8721	C_15_H_13_O_6_	Catechin (std)
3	7.22	289.0693	289.0712	−6.6	245.0787/137.0222/125.0238	C_15_H_13_O_6_	Epicatechin (std)
4	9.98	479.0845	479.0826	4.0	317.0249/316.0233/287.0161/271.0255	C_21_H_19_O_13_	Myricetin-3-*O*-galactoside
5	10.21	729.1411	729.1456	−6.2	577.1219/451.1033/441.0815/407.0768/289.0728/287.0542	C_37_H_29_O_16_	Procyanidin B1 monogallate
6	10.60	493.0612	493.0618	−1.2	317.0285/287.0196/178.9975	C_21_H_17_O_14_	Myricetin-*O*-glucuronide
7	10.87	441.0815	441.0822	−1.6	289.0693/169.0157/125.0238	C_22_H_17_O_10_	Epicatechin-3-*O*-gallate (std)
8	11.11	567.2066	567.2078	−2.1	341.1396/326.11326/160.8430	C_27_H_35_O_13_	Unknown
9	11.29	463.0859	463.0877	−3.9	317.0285/316.0233/271.0255	C_21_H_19_O_12_	Myricetin-3-*O*-deoxyhexoside
10	11.43	463.0859	463.0877	−3.9	301.0344/300.0265/271.0221	C_21_H_19_O_12_	Quercetin-*O*-hexoside 1
11	11.58	463.0859	463.0877	−3.9	301.0344/300.0265/271.0221	C_21_H_19_O_12_	Quercetin-*O*-hexoside 2
12	12.02	477.0659	477.0669	−2.1	301.0344/299.0204/271.0255	C_21_H_17_O_13_	Quercetin-3-*O*-glucuronide
13	12.38	433.0745	433.0771	−6.0	301.0344/300.0265/271.0255/255.0287	C_20_H_17_O_11_	Quercetin-*O*-pentoside 1
14	12.51	433.0745	433.0771	−6.0	301.0344/300.0265/271.0221/255.0287	C_20_H_17_O_11_	Quercetin-*O*-pentoside 2
15	17.12	331.2498	331.2484	4.2	313.2348/160.8430	C_18_H_35_O_5_	Unknown

Rt, retention time; MF, molecular formula. (std) The compound was also identified by comparing the chromatographic behavior with the authentic standard (std).

**Table 2:** In vitro antimicrobial and cytotoxic activity of the total extract from *C. cowellii* leaves.

Test Sample	Cytotoxicity (CC_50_ µg/mL)	Antimicrobial Screening (IC_50_ µg/mL)						
	MRC-5	*S. aureus*	*E. coli*	*C. albicans*	*A. fumigatus*	*C. neoformans*	*T. cruzi*	*T. brucei*
TE	>64.0	>64.0	>64.0	1.7 ± 0.6	>64.0	2.7 ± 2.0	38.3 ± 6.8	33.1 ± 0.4

TE: total extract. MRC-5: human fetal lung fibroblasts; *S. aureus*: Staphylococcus aureus; *E. coli*: Escherichia coli; *C. albicans*: Candida albicans; *A. fumigatus*: Aspergillus fumigatus; *C. neoformans*: Cryptococcus neoformans; *T. cruzi*: Trypanosoma cruzi; *T. brucei*: Trypanosoma brucei. Reference compounds: Tamoxifen (MRC-5) CC_50_ 10.5 µM; Doxycycline (*S. aureus*) IC_50_ 0.2 µM; Doxycycline (*E. coli*) IC_50_ 0.6 µM; Flucytosine (*C. albicans*) IC_50_ 0.6 µM; Econazole (*A. fumigatus*) IC_50_ 0.7 µM; Miconazole (*C. neoformans*) IC_50_ 0.2 µM; Benznidazole (*T. cruzi*) IC_50_ 3.1 µM; Suramine (*T. brucei*) IC_50_ 0.05 µM.

## Data Availability

The data presented in this study are available in Supplementary Material.

## Supplementary Materials

The following are available online, Figure S1: Molecular network of the total extract of *Coccoloba cowellii*, created with the feature-based molecular networking (FBMN) workflow on the Global Natural Products Social Molecular Networking (GNPS) web platform, Figure S2: Full MS and MS/MS spectra of compounds **1**–**15**, Figure S3: Tentative fragmentation pathways of some compounds present in the total extract of *C. cowellii* [29,55], Table S1: Library hits found in the spectra of the methanolic extract of *C. cowellii* against the GNPS database.
