# Supplementary material for: Chemical and Pharmacological Potential of Coccoloba cowellii, an Endemic Endangered Plant from Cuba
Source: Molecules. 2021 Feb 10;26(4):935. doi: 10.3390/molecules26040935 (PMC7916587; doi:10.3390/molecules26040935)
Supplement: Supplementary file 1 [file molecules-26-00935-s001.pdf]

Supplementary materials

Chemical and Pharmacological Potential of *Coccoloba cowellii*, an Endemic Endangered Plant from Cuba

Daniel Méndez [1], Julio C. Escalona-Arranz [2], Kenn Foubert [3], An Matheeussen [4], Anastasia Van der Auwera [3], Stefano Piazza [5], Ann Cuypers [6], Paul Cos [4,*] and Luc Pieters [3,*]

[1] Chemistry Department, Faculty of Applied Sciences, University of Camagüey, Carretera de Circunvalación Km 5 ½, Camagüey 74650, Cuba; daniel.mendez@reduc.edu.cu
[2] Pharmacy Department, Faculty of Natural and Exact Sciences, Oriente University, Avenida PatricioLumumba s/n, Santiago de Cuba 90500, Cuba; jcea@uo.edu.cu
[3] Natural Products & Food Research and Analysis (NatuRA), Department of Pharmaceutical Sciences, University of Antwerp, Universiteitsplein 1, BE-2610 Antwerp, Belgium; kenn.foubert@uantwerpen.be (K.F.); anastasia.vanderauwera@uantwerpen.be (A.V.d.A.)
[4] Laboratory of Microbiology, Parasitology and Hygiene (LMPH), Faculty of Pharmaceutical, Biomedical and Veterinary Sciences, University of Antwerp, Universiteitsplein 1, BE-2610 Antwerp, Belgium; an.matheeussen@uantwerpen.be
[5] Laboratory of Pharmacognosy, Department of Pharmacological and Biomolecular Sciences, University of Milan/UNIMI, IT-20133, Milan, Italy; stefano.piazza@unimi.it
[6] Centre for Environmental Sciences, Campus Diepenbeek, Hasselt University, Agoralaan Building D, BE-3590 Diepenbeek, Belgium; ann.cuypers@uhasselt.be
* Correspondence: paul.cos@uantwerpen.be (P.C.); luc.pieters@uantwerpen.be (L.P.)

Keywords: *Coccoloba cowellii*; endemic plant; UHPLC-ESI-QTOF-MS; flavonoids; antifungal; antibacterial; COX-1/2 inhibition.

Table S1. Library hits found in the spectra of the methanolic extract of *C. cowellii* against the GNPS database.

Compound name	Library class	Cosine	Shared peaks	MZErrorPPM	LibMZ
Quercetin-3-*O*-rhamnoside (Quercitrin)	Bronze	0.85	8	1	447.093
Quercetin-3-*O*-galactoside (Hyperoside)	Bronze	0.80	7	0	463.088
Quercetin-3-*O*-arabinoside (Avicularin)	Bronze	0.72	6	0	433.078
Quercetin-3-*O*-glucuronide (Miquelianin)	Bronze	0.84	6	1	477.067
Quercetin 3-(2-galloylglucoside)	Bronze	0.73	6	37	615.099
Myricetin-3-*O*-pentoside	Bronze	0.85	6	10	449.067
Myricetin-3-*O*-galactoside	Bronze	0.93	9	2	479.083
4′-*O*-Methylmyricetin-3-*O*-rhamnoside (Mearnsitrin)	Gold	0.83	8	93	477.104
Procyanidin B1	Bronze	0.81	11	1	577.136
Procyanidin B2	Bronze	0.71	9	14	575.108
Catechin-3-*O*-gallate	Bronze	0.81	8	2	441.083
Epicatechin-3-*O*-gallate	Bronze	0.71	8	10	487.088

MZErrorPPM: ppm error with the spectral library match, LibMZ: *m*/*z* value of the spectral library match.

Figure S1. Molecular network of the total extract of *Coccoloba cowellii*, created with the Feature-Based Molecular Networking (FBMN) workflow on the Global Natural Products Social (GNPS) molecular networking web-platform (The molecular networking job can be publicly accessed at https://gnps.ucsd.edu/ProteoSAFe/status.jsp?task=a2f9e6e25ca64043a36a3d2fb09270c5)

Figure S2. Full MS and MS/MS spectra of compounds 1-15.

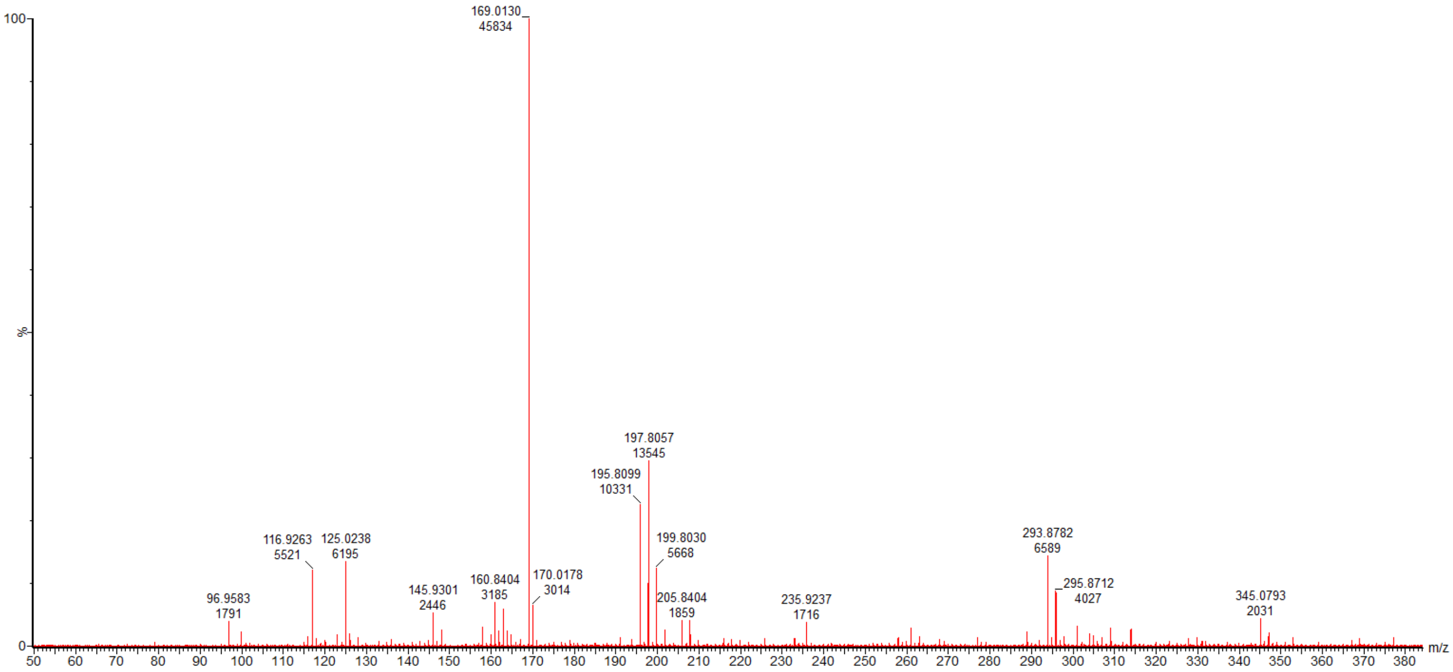

a) Peak 1 MS spectrum, Rt 2.03 min

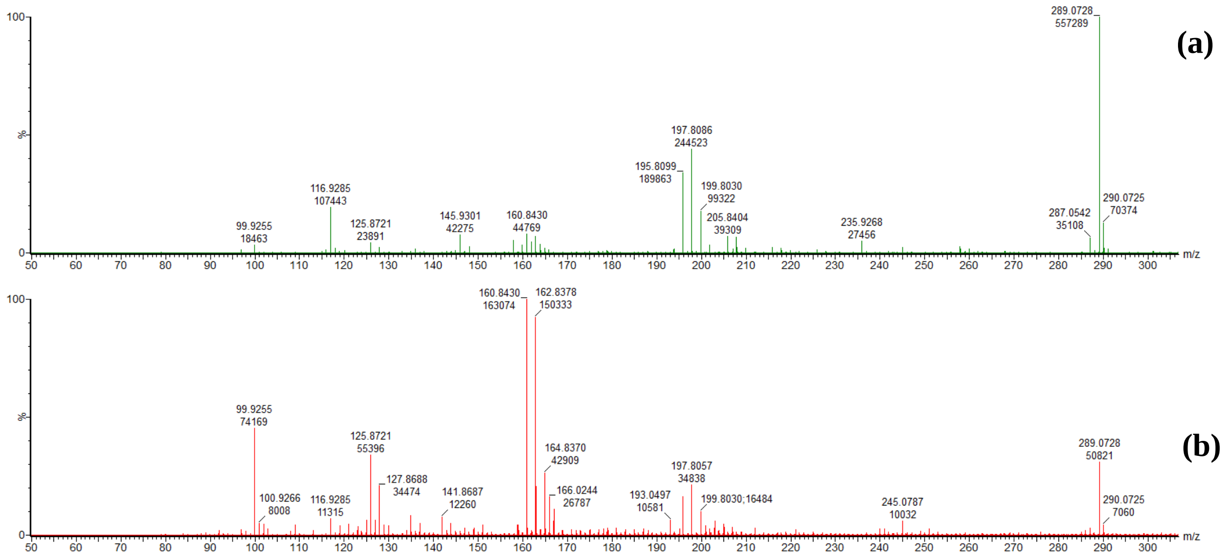

b) Peak 2 [(a) MS spectrum and (b) MS/MS spectrum], Rt 6.04 min

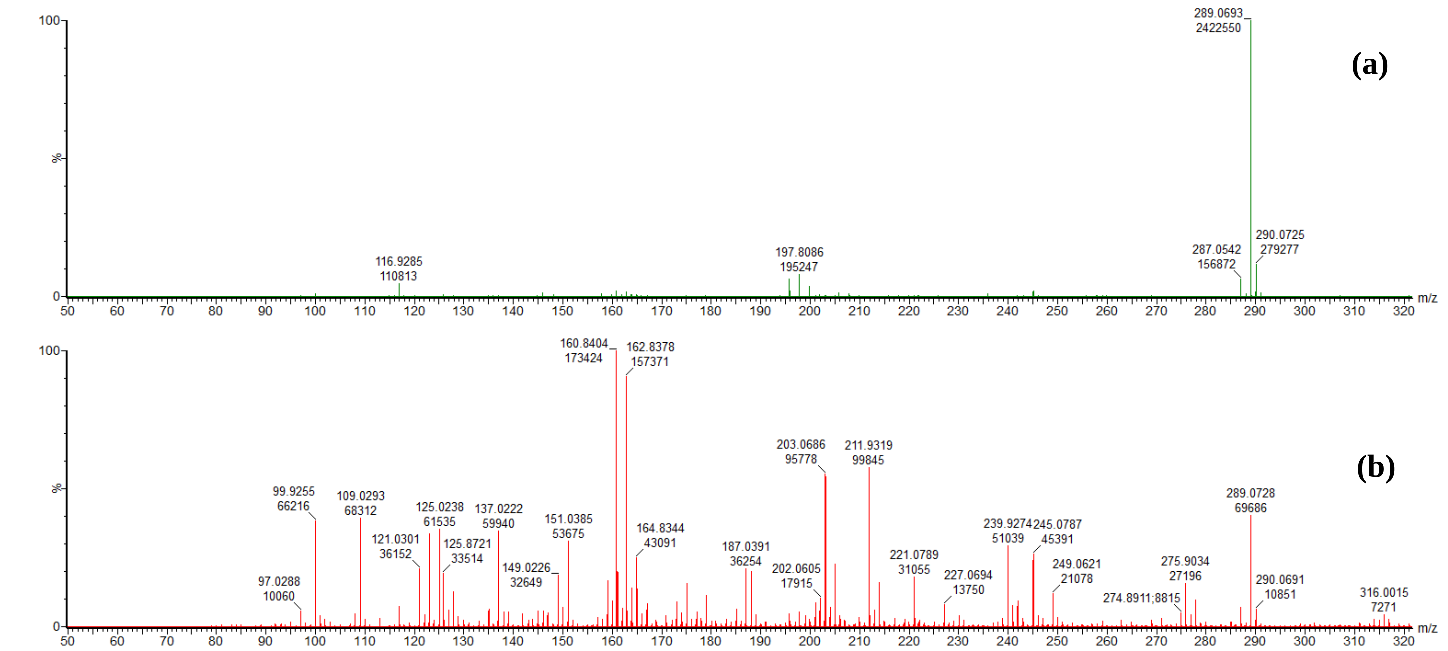

c) Peak 3 [(a) MS spectrum and (b) MS/MS spectrum], Rt 7.22 min

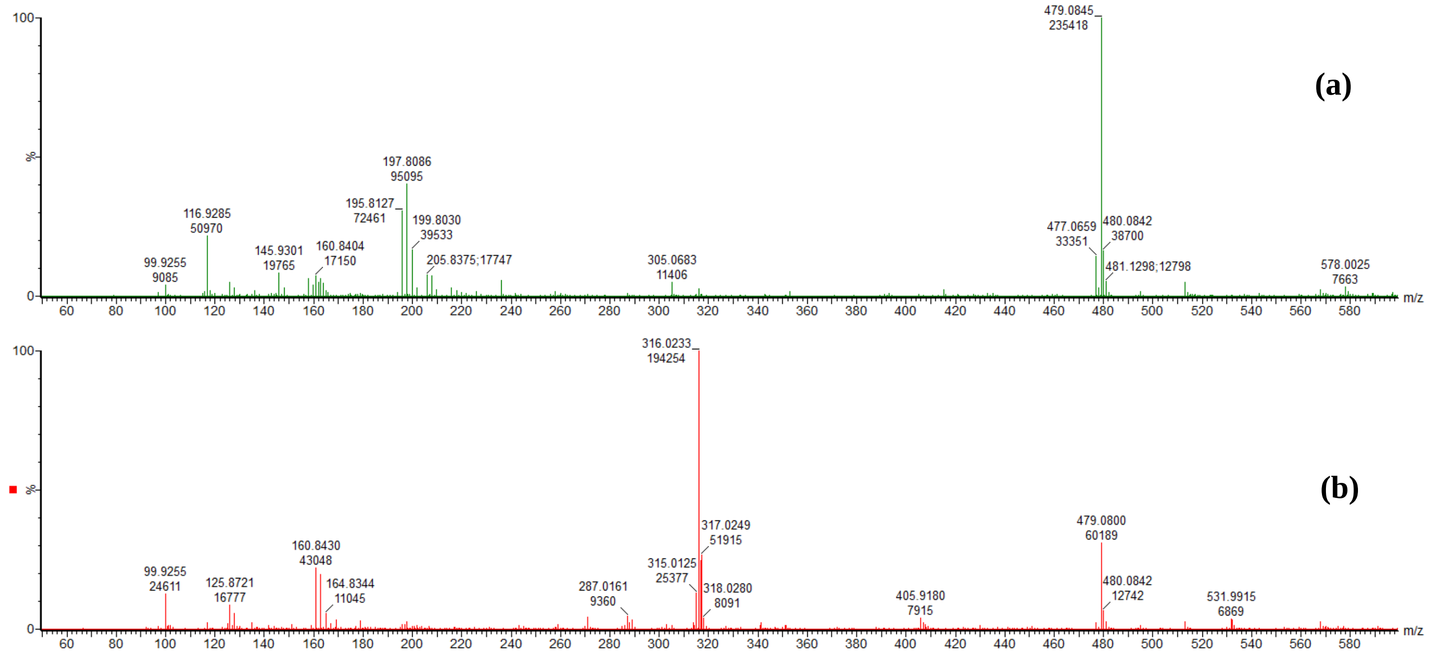

d) Peak 4 [(a) MS spectrum and (b) MS/MS spectrum], Rt 9.98 min

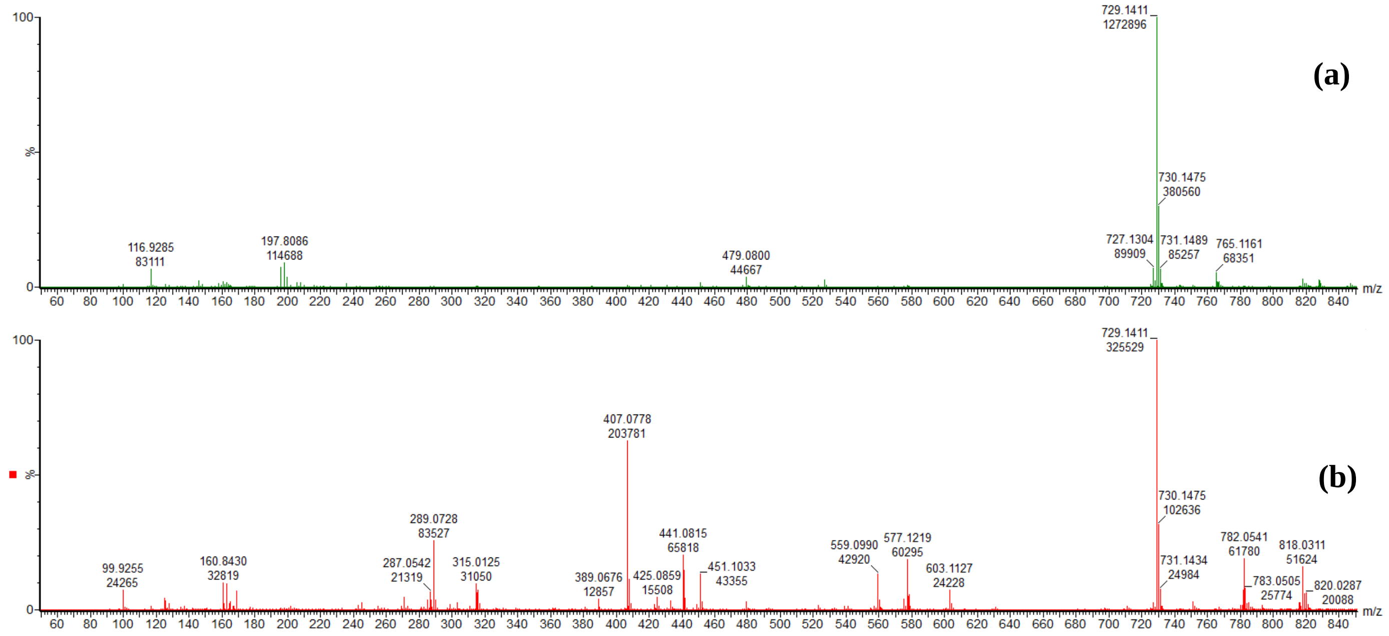

e) Peak 5 [(a) MS spectrum and (b) MS/MS spectrum], Rt 10.21 min

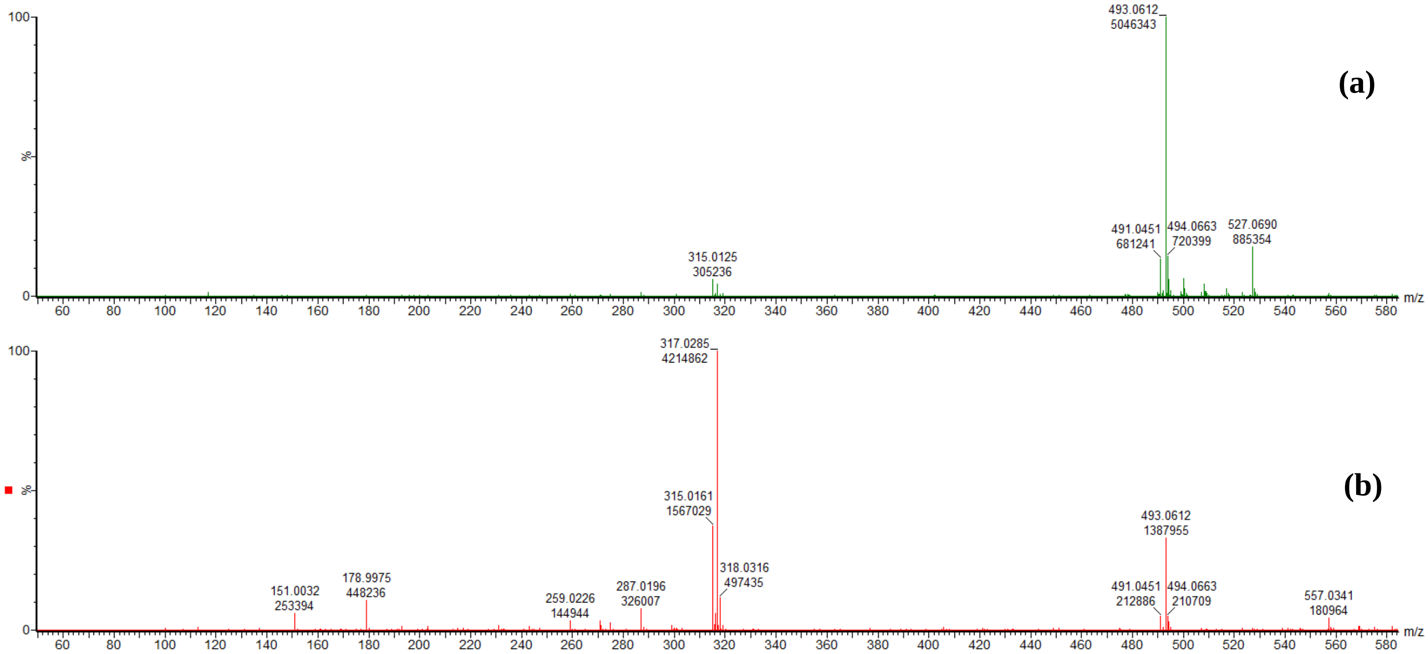

f) Peak 6 [(a) MS spectrum and (b) MS/MS spectrum], Rt 10.60 min

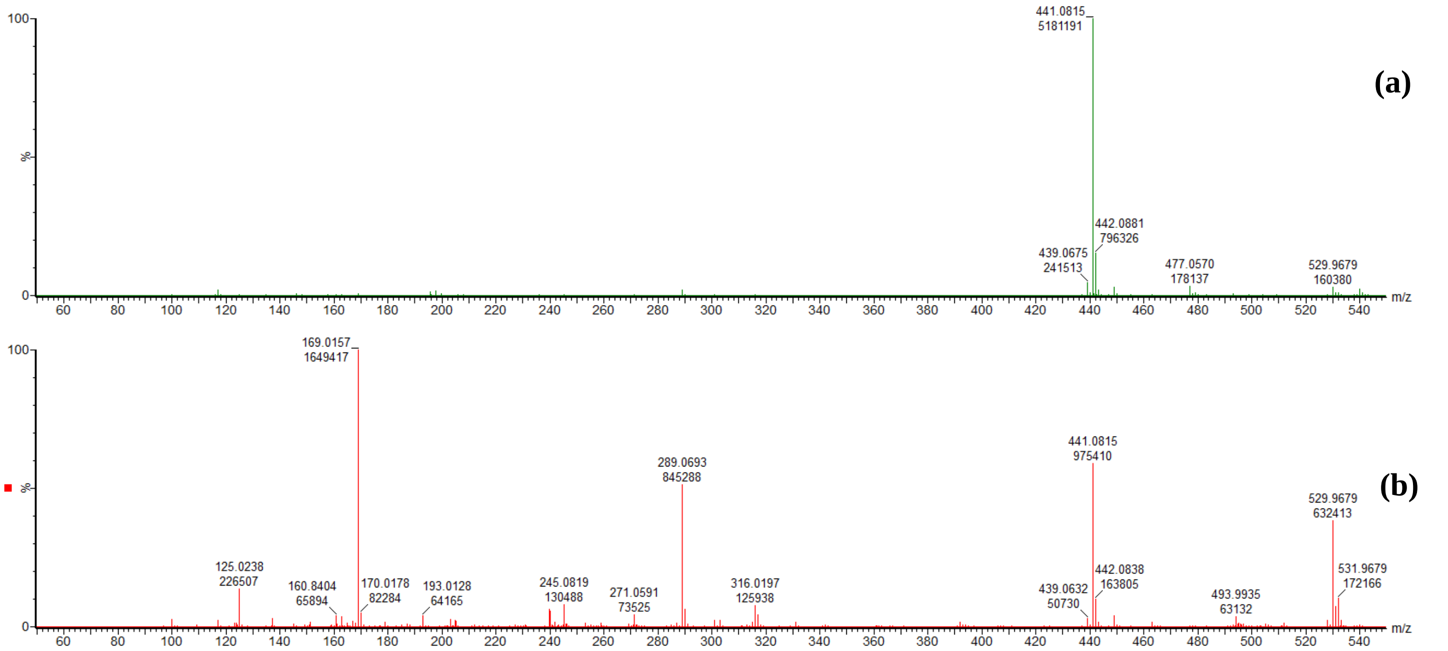

g) Peak 7 [(a) MS spectrum and (b) MS/MS spectrum], Rt 10.87 min

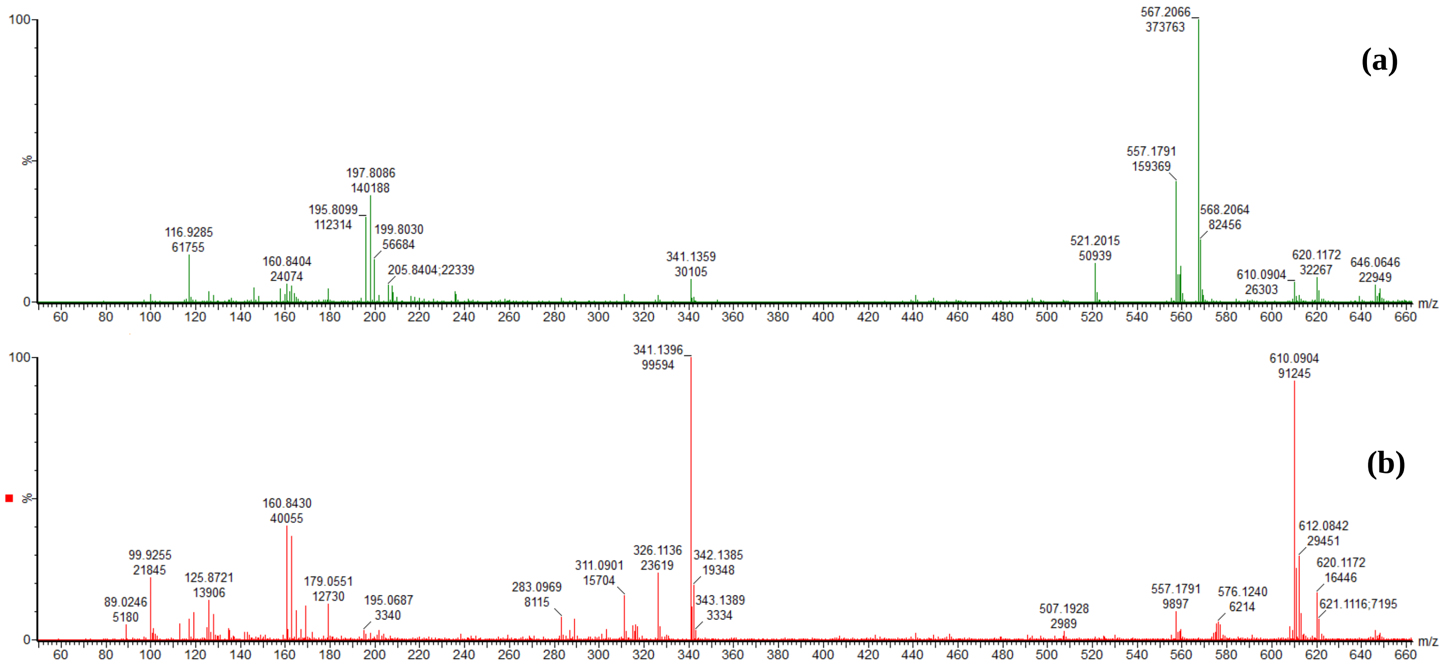

h) Peak 8 [(a) MS spectrum and (b) MS/MS spectrum], Rt 11.11 min

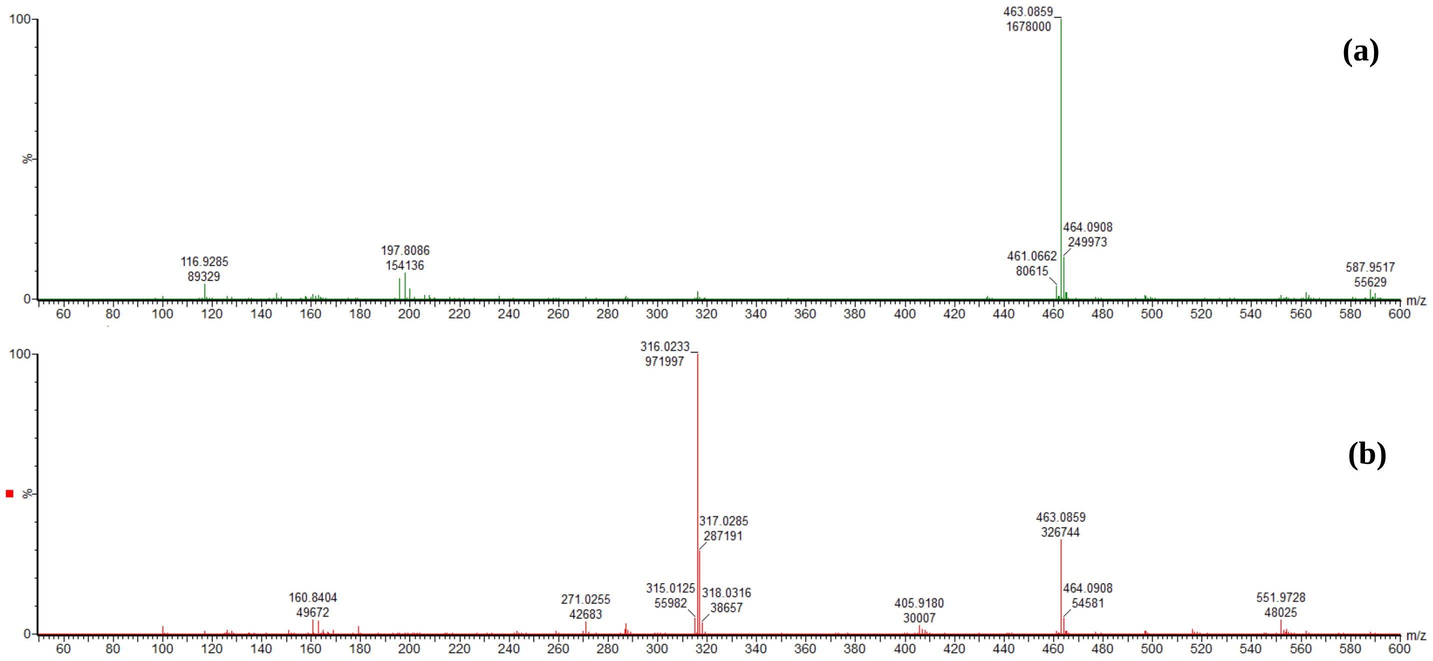

i) Peak 9 [(a) MS spectrum and (b) MS/MS spectrum], Rt 11.29 min

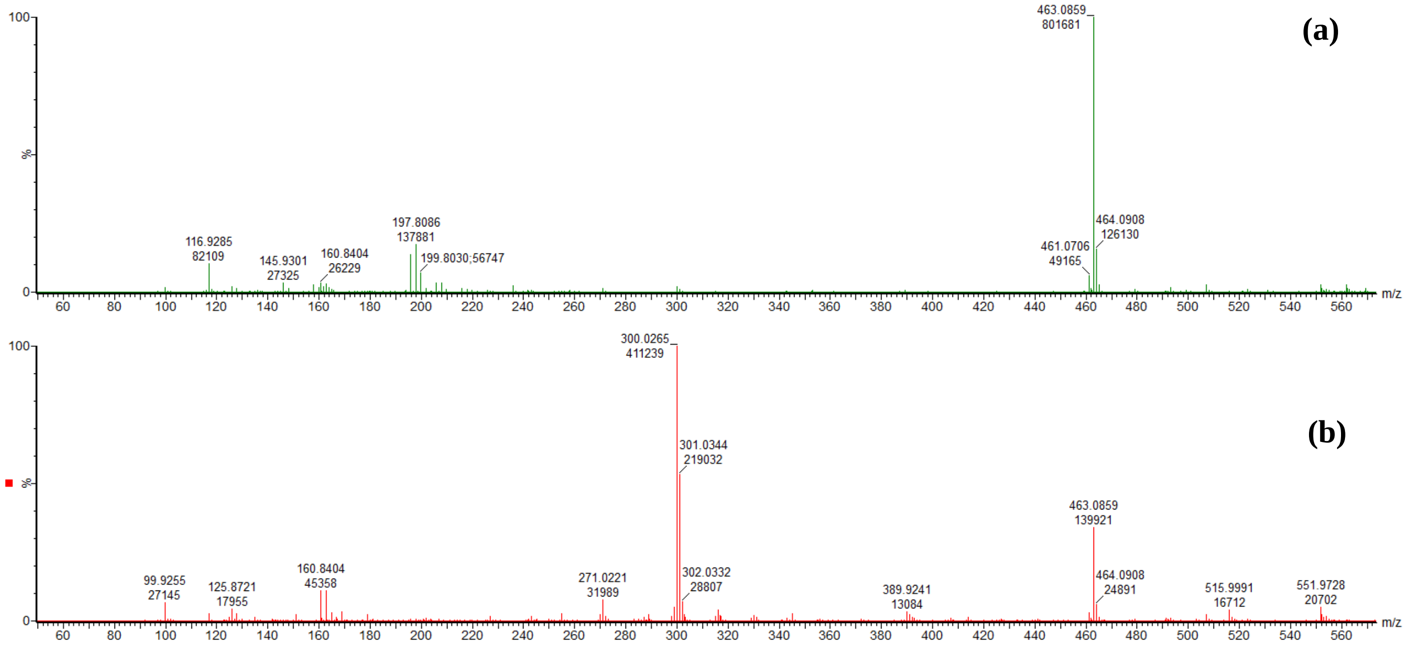

j) Peak 10 [(a) MS spectrum and (b) MS/MS spectrum], Rt 11.43 min

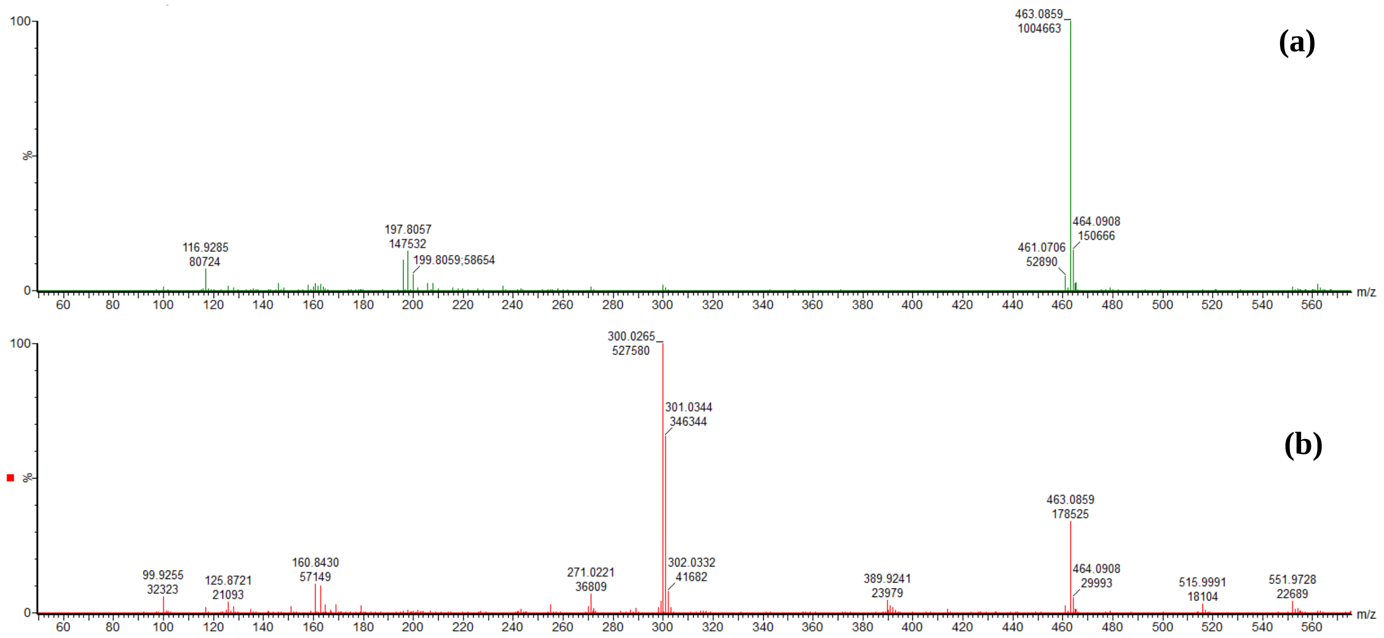

k) Peak 11 [(a) MS spectrum and (b) MS/MS spectrum], Rt 11.58 min

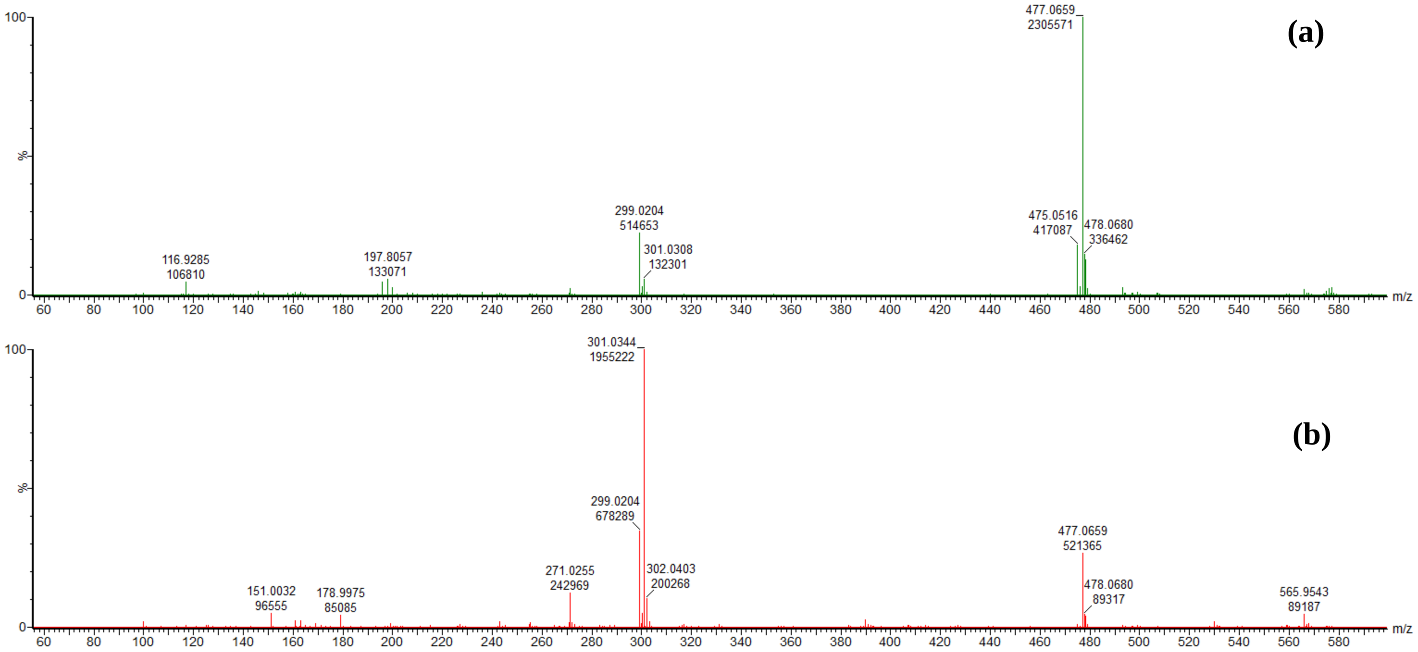

l) Peak 12 [(a) MS spectrum and (b) MS/MS spectrum], Rt 12.02 min

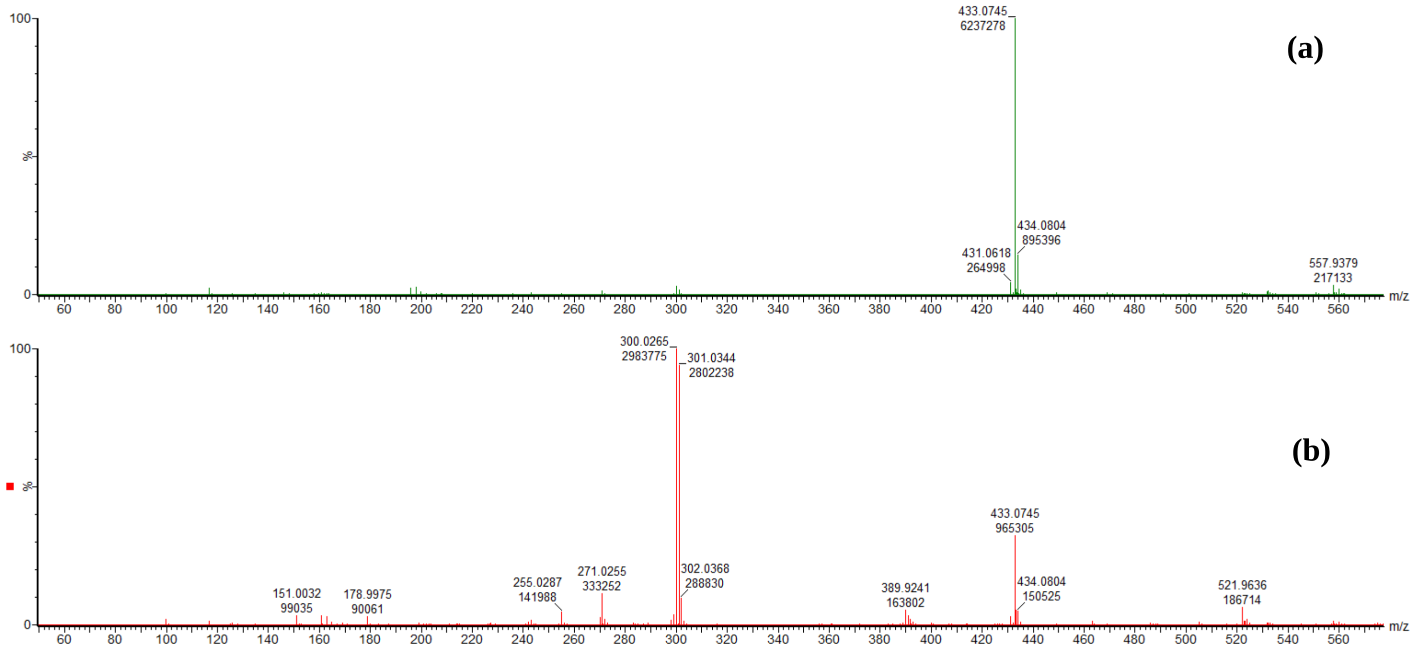

m)   Peak 13 [(a) MS spectrum and (b) MS/MS spectrum], Rt 12.38 min

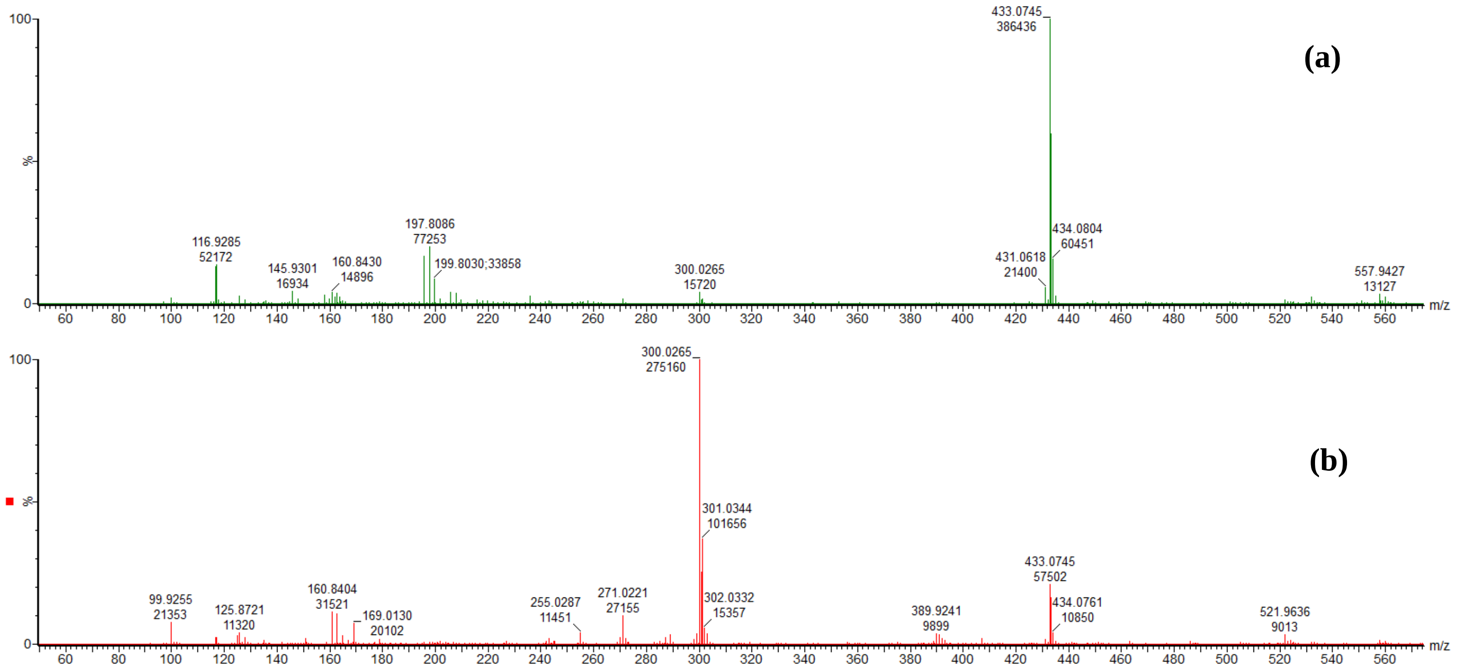

n)   Peak 14 [(a) MS spectrum and (b) MS/MS spectrum], Rt 12.51 min

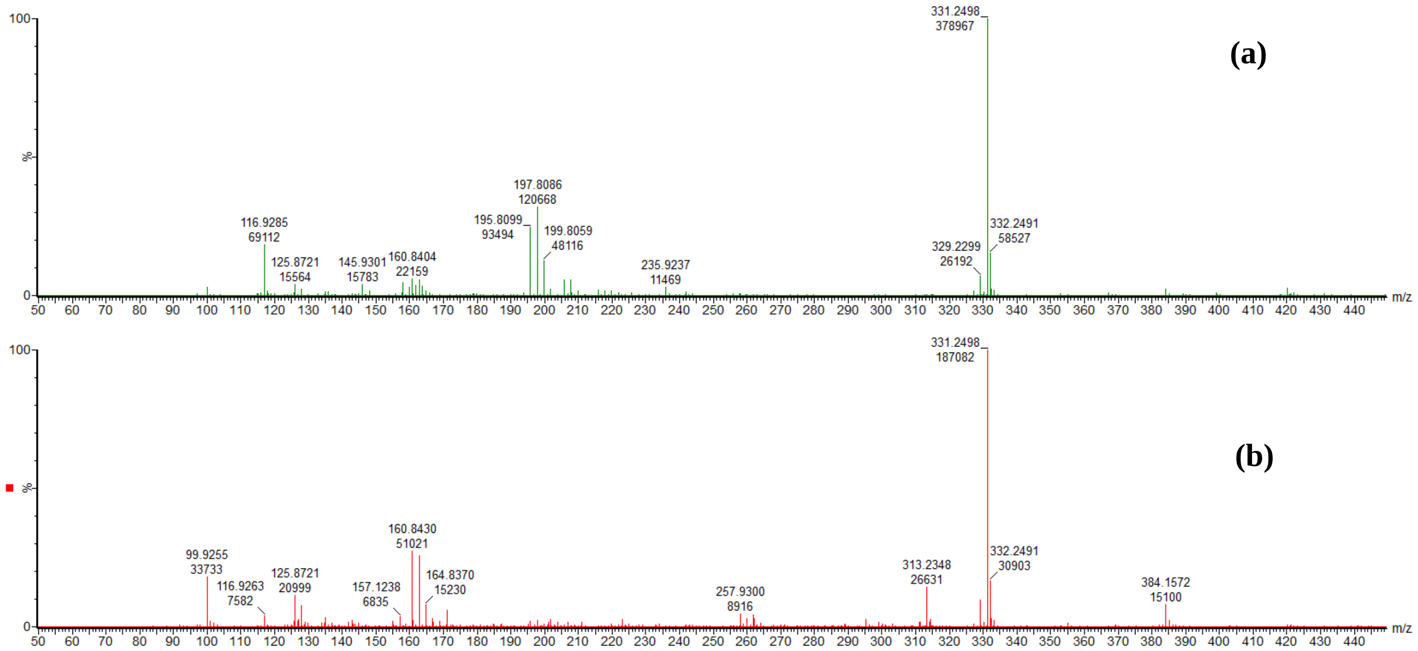

o) Peak 15 [(a) MS spectrum and (b) MS/MS spectrum], Rt 17.12 min

Figure S3. Tentative fragmentation pathways of some compounds present in the total extract of *C. cowellii*.

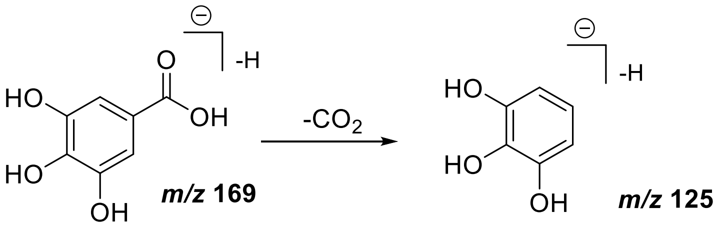

a) Peak 1, Rt 2.03 min, tentative identification: Gallic acid

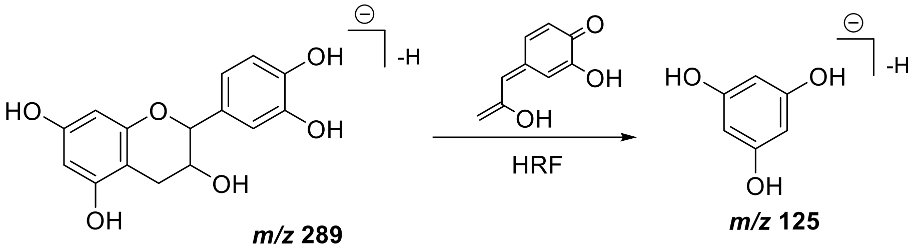

b) Peak 2, Rt 6.04 min, tentative identification: Catechin. Hypothetical fragmentation pattern taken from Callemien and Collin, 2008 [1].

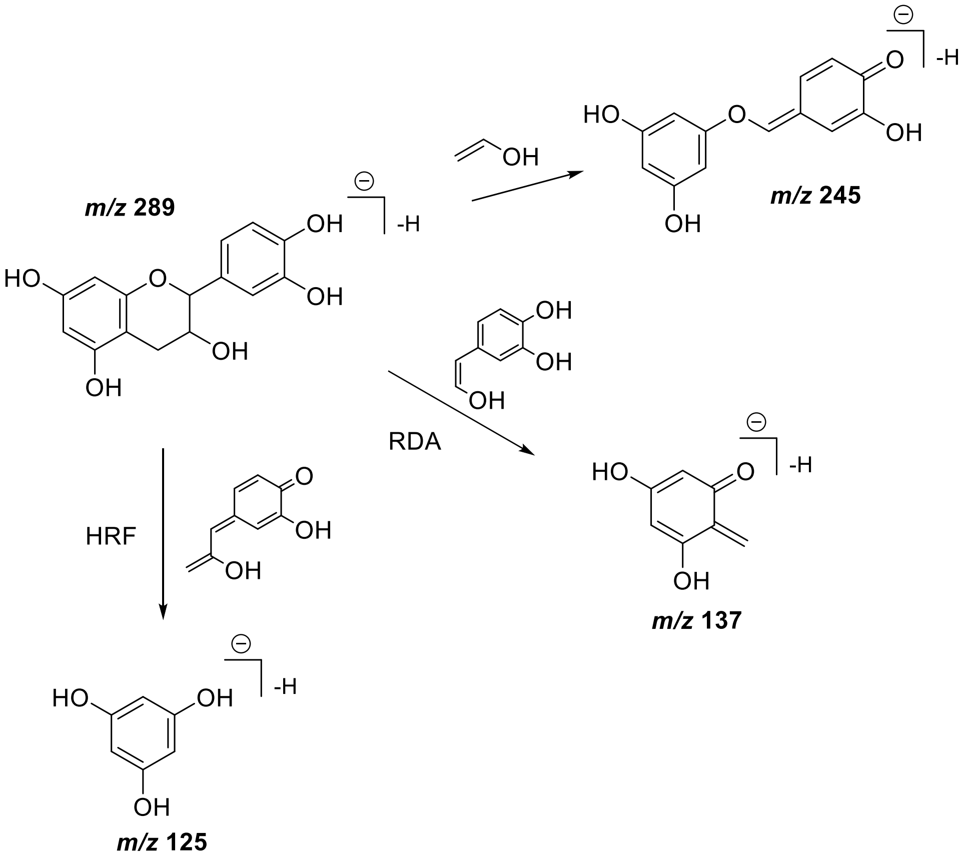

c) Peak 3, Rt 7.22 min, tentative identification: Epicatechin. Hypothetical fragmentation pattern taken from Callemien and Collin, 2008 [1].

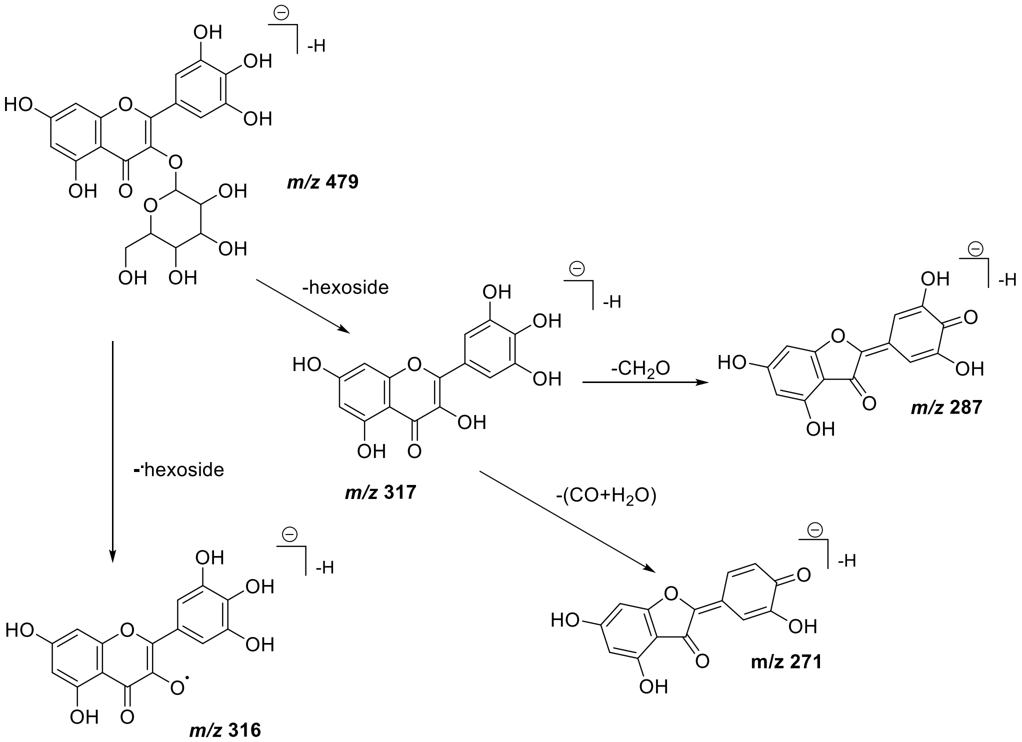

d) Peak 4, Rt 9.98 min, tentative identification: Myricetin-3-O-galactoside. Hypothetical fragmentation pattern taken and adapted from Li et al., 2016 [2].

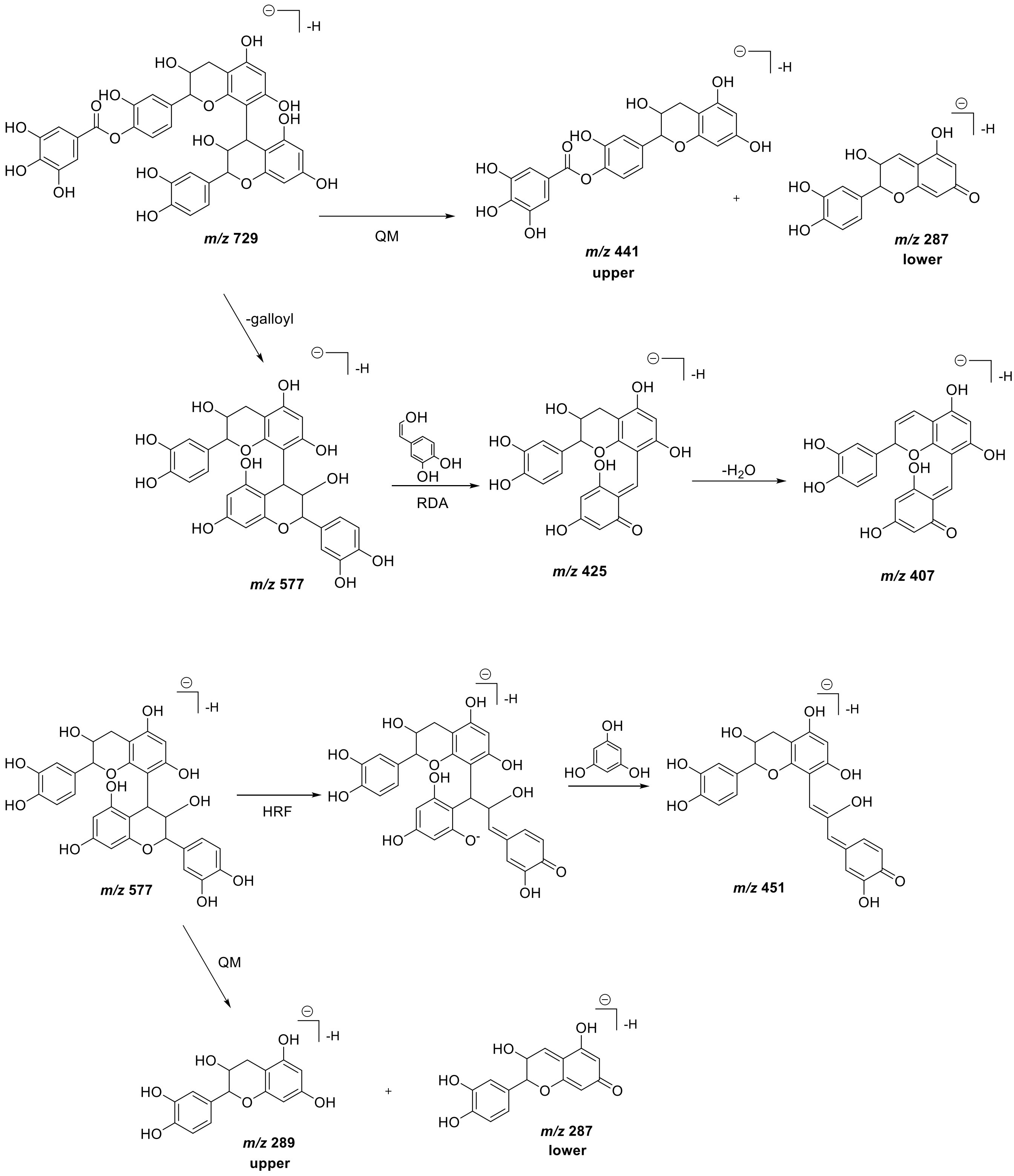

e) Peak 5, Rt 10.21 min, tentative identification: Procyanidin B1 monogallate (position of the galloyl substituent is arbitrary). Hypothetical fragmentation pattern taken from Callemien and Collin, 2008 [1].

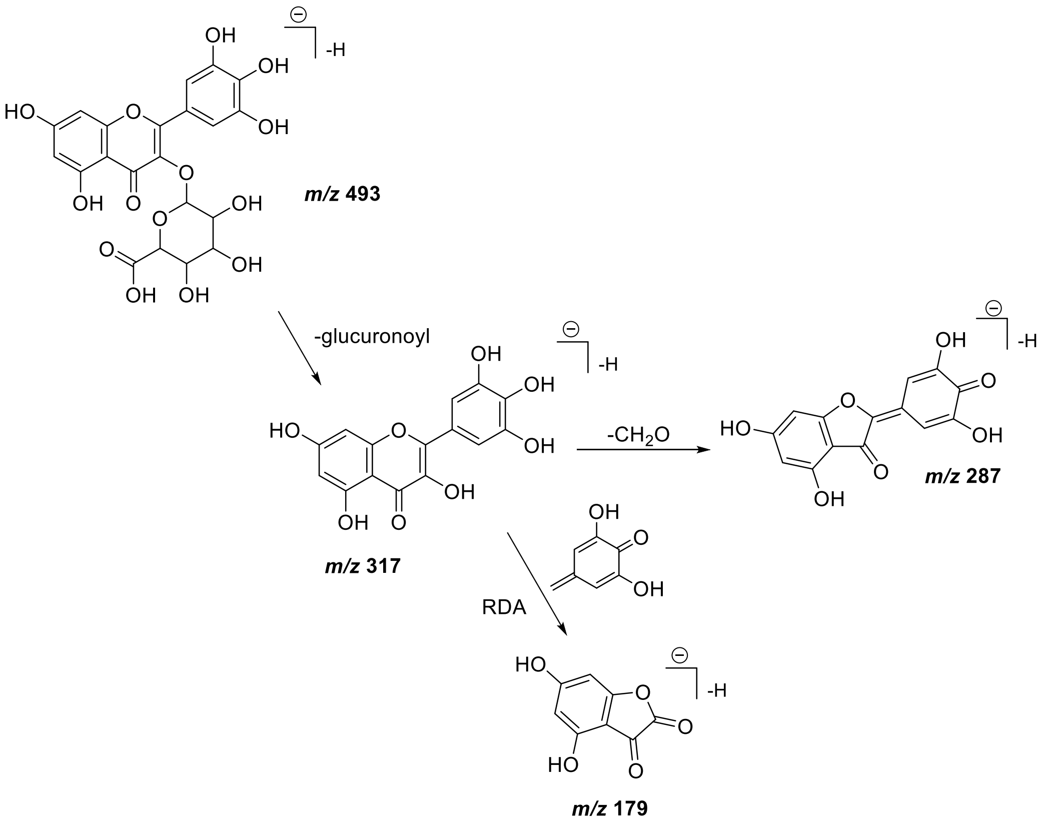

f) Peak 6, Rt 10.60 min, tentative identification: Myricetin-3-O-glucuronide. Hypothetical fragmentation pattern taken and adapted from Li et al., 2016 [2].

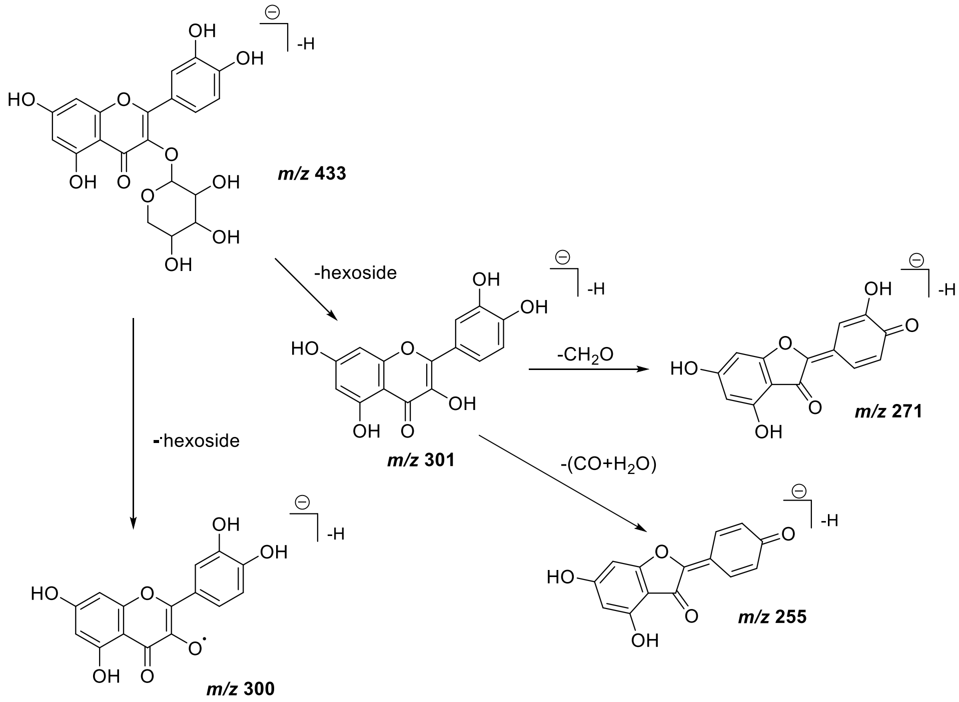

g) Peak 13, Rt 12.38 min, tentative identification: Quercetin-O-pentoside 1. (position of hexoside substituent is arbitrary). Hypothetical fragmentation pattern taken from Li et al., 2016 [2].

1. Callemien, D.; Collin, S. Use of RP-HPLC-ESI(-)-MS/MS to differentiate various proanthocyanidin isomers in lager beer extracts. *J. Am. Soc. Brew. Chem.* **2008**, *66*, 109–115, doi:10.1094/ASBCJ-2008-0215-01.
2. Li, Z.H.; Guo, H.; Xu, W. Bin; Ge, J.; Li, X.; Alimu, M.; He, D.J. Rapid Identification of Flavonoid Constituents Directly from PTP1B Inhibitive Extract of Raspberry (Rubus idaeus L.) Leaves by HPLC-ESI-QTOF-MS-MS. *J. Chromatogr. Sci.* **2016**, *54*, 805–810, doi:10.1093/chromsci/bmw016.
